# Mutualistic networks emerging from adaptive niche-based interactions

**DOI:** 10.1038/s41467-020-19154-5

**Published:** 2020-10-29

**Authors:** Weiran Cai, Jordan Snyder, Alan Hastings, Raissa M. D’Souza

**Affiliations:** 1 Department of Computer Science, University of California at Davis, Davis, CA 95616 USA; 2 Complexity Sciences Center, UC Davis, Davis, CA 95616 USA; 3 Department of Mathematics, UC Davis, Davis, CA 95616 USA; 4 Department of Environmental Science and Policy, UC Davis, Davis, CA 95616 USA; 5 Santa Fe Institute, Santa Fe, NM 87501 USA

**Keywords:** Network topology, Applied mathematics

## Abstract

Mutualistic networks are vital ecological and social systems shaped by adaptation and evolution. They involve bipartite cooperation via the exchange of goods or services between actors of different types. Empirical observations of mutualistic networks across genres and geographic conditions reveal correlated nested and modular patterns. Yet, the underlying mechanism for the network assembly remains unclear. We propose a niche-based adaptive mechanism where both nestedness and modularity emerge simultaneously as complementary facets of an optimal niche structure. Key dynamical properties are revealed at different timescales. Foremost, mutualism can either enhance or reduce the network stability, depending on competition intensity. Moreover, structural adaptations are asymmetric, exhibiting strong hysteresis in response to environmental change. Finally, at the evolutionary timescale we show that the adaptive mechanism plays a crucial role in preserving the distinctive patterns of mutualism under species invasions and extinctions.

## Abstract (web summary)

Nested and modular patterns are vastly observed in mutualistic networks across genres and geographic conditions. Here, the authors show a unified mechanism that underlies the assembly and evolution of such networks, based on adaptive niche interactions of the participants.

## Introduction

Mutualism, the interaction that joins actors providing reciprocal benefits or services, has a pivotal role in nature and human society^1–5^. Many mutualistic systems have formed through self-organization rather than through explicit engineering. Still, common structural features describing the relationship of the actors have been observed pervasively in mutualistic systems across many scales and contexts, suggesting a unifying underlying mechanism. Such relations can be captured by a bipartite cooperation network among two distinct guilds of actors where edges between guilds represent a form of cooperation such as the “barter” between plants and pollinators or an industrial partnership between designers and contractors^1–7^. The most distinctive features of these networks are the modular and nested patterns that are consistently overexpressed compared with their randomized counterparts^4,6,7^. A modular organization implies that most mutualistic links can be contained in several clusters, whereas a nested structure implies that more often than not the partners of one species of a lower degree (specialist) are a subset of the partners of another species of a higher degree (generalist).

Potential origins of these pervasive structures have been studied by various approaches. From the perspective of static theories, the network structure is interpreted as a consequence of the appropriate matching of traits or abundances^4,8–14^. Among them, the niche model of Saavedra et al.^4^, which follows along the line of Williams and Martinez^8,9^, demonstrates a host of mutualistic network structures as proper trait relations in a concise way. Beyond studies of static structural properties, dynamic theories aim to explain the emergence of the network structure and predict its future development. Such theories have been developed for numerous problems from food web assembly to trophic structure to specialization trends^15–17^. Although mutualistic partnerships are also known to be adaptive in nature in both ecological and socio-economic contexts^2,4,18,19^, most dynamic theories for mutualism focus partially on nestedness^13,20–22^, leaving modularity unexplored. However, the strong correlation between the nestedness and modularity observed in empirical networks suggests they have a common origin. Thus, having a unified, dynamical process that elucidates how both nested and modular patterns emerge in an integrated manner is highly desired.

Here, we propose an adaptive niche model, incorporating the quintessential concept of niches and the adaptive dynamics of connection. An optimal partnership structure exhibiting both nestedness and modularity emerges from a unified optimization mechanism accounting for the participants’ niche relations and population distribution^2,18,19,23^. Positive feedback of local advantages plays a central role in the co-emergence of the patterns. The dyadic measures of the assembled networks are correlated and comparable to those observed in empirical networks. Key dynamical properties are revealed at different timescales. At the ecological timescale, we show the bidirectional role of mutualism on network stability: enhancing mutualism can either stabilize or destabilize the network, depending primarily on the intensity of competition. We also demonstrate that network adaptation is asymmetric, exhibiting a prominent hysteresis in response to environmental change. Accidents of environmental history may thus be “frozen” in the network structure. Finally, at the evolutionary timescale we show that the adaptive behavior leads to different resilience of generalist and specialist nodes in the presence of species invasions and extinctions that is crucial for preserving the patterns of mutualism during evolution.

## Results

### Adaptive niche model

We study network assembly using the ecological concept of niche-based interactions, which may extend to a broad range of non-biological contexts where mutual benefits exist such as bipartite partnerships among socio-economic organizations. We concentrate on a pair of fundamental characteristics of each actor (e.g., species, company, or social organization): first its niche, being the living or operating range, and second its fitness, being the abundance or operating status.

**Fig. 1:**
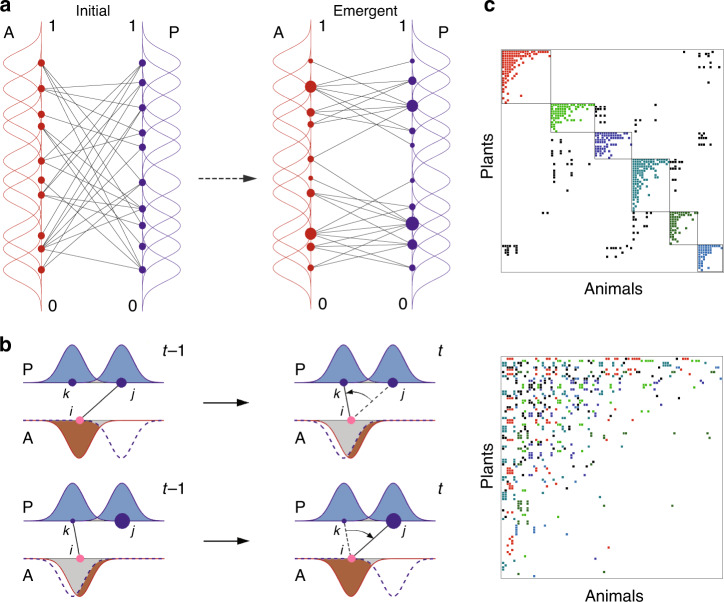
Pattern formation from adaptive niche-based interactions. **a** Adaptation of niche relation and demographic distribution. An example network of 20 species in mutually interacting guilds (A and P) develops from a random relation (left) to a stable partnership (right). Each species *i* possesses a pair of characteristics: a niche profile (Gaussian function *H*_*i*_(*s*)) randomly scattered on the niche axis, and an individual species abundance (represented by a disk of proportional size). The species abundances are governed by the generalized Lotka-Volterra dynamics (Eq. 5a, Eq. 5b), where the coupling strengths are determined by the proximity of interacting specific niches across the guilds for mutualism and within the guilds for competition. **b** Rewiring and updating niche proximity. A species rewires to a randomly selected new partner species with updated niche proximity. The rewiring is accepted if the individual species abundance increases and rejected otherwise. The example in the upper panel demonstrates the rewiring of a node *i* from a partner *j* with a slightly higher abundance to a new one *k* with a larger niche proximity, *H*_*ij*_ → *H*_*ik*_. In contrast, the lower panel shows that a node rewires to a new partner with a smaller niche proximity but a significantly higher abundance. The gray area represents the niche proximity. **c** Network patterns in steady state. An assembled network is shown for example, with 100 species in each guild, simulated for *C*_0_ = 0.058 and (Ω_*m*_, Ω_*c*_) = (0.10, 0.05), with significantly higher nestedness and modularity (NODF = 0.1666, Q = 0.6207) than the randomized networks (*P* ≤ 0.0001; two-sided *t* test). The adjacency matrix is reordered to express the modular (upper panel) and nested (lower panel) patterns of the same network, with the colors indicating the module memberships.

For concreteness we explain the model in the context of pollination. Consider a collection of species in two distinct guilds (denoted *P* and *A* in analogy with plants and animal pollinators) that are involved in mutualism with selected partners in the opposite guild and competition with all rivals in their own guild (Fig. 1a). The niche profile of a species can be generally formulated by a Gaussian function *H*_*i*_(*s*), representing its statistical distribution on a one-dimensional niche axis^24,25^. We define niche proximity *H*_*ij*_ as the joint occupation probability of a pair of interacting actors on the niche axis^26,27^

1 $${H}_{ij}=\int{H}_{i}(s){H}_{j}(s)ds,$$

For within-guild interactions, niche proximity considers habitat niches capturing trait similarity of rival species in competition with one another (e.g., for similar nesting sites or soil conditions). For cross-guild interactions, niche proximity instead considers partner niches capturing trait complementarity of mutualistic partners.

The species abundance *n*_*i*_ follows coupled dynamics, where the coupling strength is proportional to the niche proximity {*H*_*ij*_} of interacting species

2 $$\frac{d{n}_{i}}{dt}={F}_{i}(\{{n}_{i}\},\{{H}_{ij}\})\quad (i=1\ldots M),$$

where *M* is the total number of species. We use the generalized Lotka-Volterra dynamics, where the mutualistic interactions are described by Holling type II functional response^28^. This implies that we track the mean fitness of individual species, regardless of intraspecific difference. The mutualistic and competitive interaction strengths are encoded in the coupling matrices {*γ*_*ij*_} and {*β*_*ij*_}, respectively (see Methods).

We assume that the niche relations among the species change adaptively to maximize the fitness of individual species^26^. At fixed time intervals, a randomly chosen species attempts to rewire to a different mutualistic partner and the niche proximity is updated (Fig. 1b). The rewiring is accepted if the individual species abundance increases and rejected otherwise^20,21^. In addition, a rewiring probability is used to reflect the situation that link removal from a specialist is harder than from a generalist (see Methods). The population dynamics is relaxed to an equilibrium between the rewiring attempts.

### Emergent structures

**Fig. 2:**
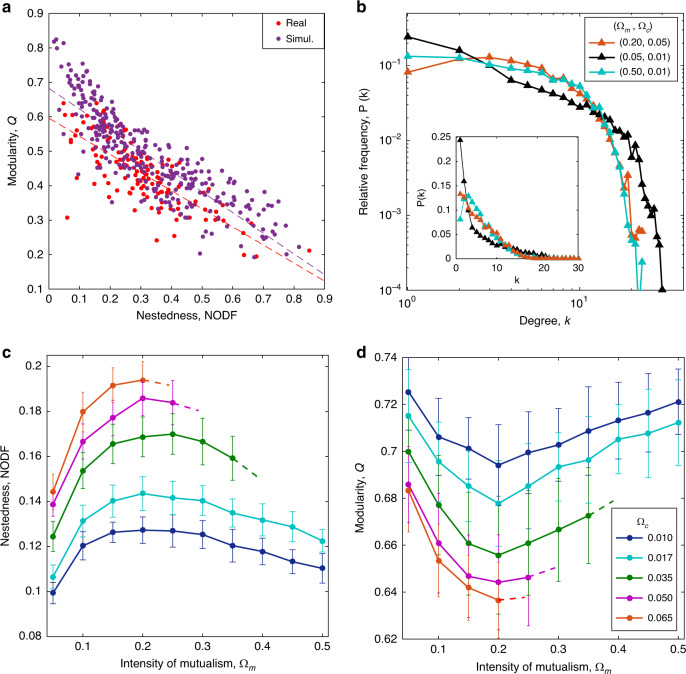
Assembled network structure. **a** Systematic comparison of dyadic structural measures of real and generated networks. The dyadic measures (nestedness NODF, modularity Q) of an ensemble of 300 generated networks (red) show a significant overlap with those of the 144 empirical networks (blue) from the Web of Life data set and exhibit a similar negative correlation (dashed lines). For each model network, the numbers of animal and plant species and the connectance are taken from a randomly chosen empirical network, covering a large variety of network sizes (ranging from 21 to 1500) and aspect ratios (ranging from 1 to 9.8). The interaction intensities (Ω_*m*_, Ω_*c*_) are chosen uniformly at random from [0.01, 0.30] × [0.01, 0.30] and niche width *σ* from [0.01, 0.50]. **b** Emergent degree distribution showing a truncated power law for relatively low modularity (shown for (Ω_*m*_, Ω_*c*_) = (0.20, 0.05)) and a single-peaked distribution for relatively high modularity (shown for (Ω_*m*_, Ω_*c*_) = (0.05, 0.01) and (0.50, 0.01)). The sizes of both guilds are *M*_*A*_ = *M*_*P*_ = 100 and connectance *C*_0_ = 0.058 (same for all panels). **c**, **d** Structural measures versus interaction intensities. The nestedness and modularity measures show a convex and concave dependence on the intensity of mutualism Ω_*m*_, respectively; whereas the family of colored curves show that enhancing the competition intensity Ω_*c*_ contributes positively to NODF and negatively to Q. Data are obtained from 50 simulation runs and presented as mean values  ±  SD. The dashed tips represent infeasible equilibria. Note that different combinations of (Ω_*m*_, Ω_*c*_) can generate networks with the same (NODF, Q) values.

Our numerical simulations generate an ensemble of extensive network structures, exhibiting a broad range of nestedness and modularity (Fig. 2a). In all realizations, we start from a randomly connected bipartite mutualistic network ${\{{\gamma }_{ij}\}}_{t = 0}$ with a specified connectance *C*_0_. The center positions $\bar{{s}_{i}}$ of niche functions are sampled uniformly at random from the niche axis. We use such random initial structures as the “starting point” of assembly, representing arbitrary partnerships in the beginning. We demonstrate that structured networks can be self-assembled irrespective of such initial arbitrariness.

The bipartite niche relations change until reaching a state where all macroscopic structural and demographic measures remain approximately constant with time, which we refer to as the steady state. We measure the nestedness with the metric NODF^29^ and the modularity with the leading eigenvector algorithm^30^. The modules are robust in that, except for a few peripheral ones, most of the species are partitioned persistently to only one of the modules once the evolved steady state is reached (see Supplementary Fig. 4a, b for the metric of module stationarity). In this sense, the entire community has settled into a macroscopic order after exploring a landscape of numerous possibilities of niche relations^31^.

Notably, the generated nested and modular measures are statistically comparable with those of the empirical ecological networks from the Web of Life data set^32^, which range over a large variety of geographical factors and constituents. As shown in Fig. 2a, the band of dyadic measures (NODF, Q), simulated with the exterior characteristics (size, connectance, aspect ratio) of the empirical networks, cover almost the full range of possible structures and also regenerate a similar negative correlation between NODF and Q as exhibited in the empirical ensemble (note that there is still a systematic difference; see Supplementary Note 5 for the possible cause). The correlation indicates that the two measures cannot be determined independently: one can find networks with very high nestedness (modularity) and very low modularity (nestedness), but not likely with both being high or low. In the case of both nontrivial nestedness and modularity (significantly higher than for the null model), modules may emerge with embedded nested structures^33,34^, as exemplified in Fig. 1c.

The degree distribution, describing the heterogeneity in the numbers of partners per species, transforms from a Poisson distribution of the initial random network to a stable distribution at the steady state. The stable distribution varies with the modularity (Fig. 2b): a truncated power law is present for a relatively low modularity (corresponding to relatively large module sizes). With the increase of modularity, it converts to a narrower single-peaked distribution owing to the fact that species contained within a smaller-sized module tend to possess comparable numbers of partners.

### Cumulative advantage

Heuristically, the nested and modular patterns are formed coherently through a positive feedback of local advantages. Driven by the incentive of increasing individual species fitness, a preponderance in the abundance of a certain species tends to attract more partner species within a larger range of niche proximity, which in turn enhance its own abundance (as indicated by the positive correlations of species abundance, degree and broadness of partnership in Supplementary Fig. 5c, d). The modular and nested structure is hence formed by the aggregation of links around separate hubs as “seeds” consisting of generalists (see network assembly in Supplementary Fig. 1). Both patterns are thus formed along the same path of development and exhibit a negative correlation. This optimization dynamics belongs to a broad class of localized preferential attachment processes, whereby “the rich get richer”, but under the local constraints on the potential linkage^35–37^. This effect known as cumulative advantage^38,39^, prevailing in socio-economic systems, thus also underlies the formation of mutualistic relationship.

Inadvertently this process can also reduce competition. While direct competition (associated with habitat niches) and direct mutualism (associated with partner niches) are explicit in the population dynamics, indirect mutualism can also emerge between competing species. Competitors in close habitat niches can affect each other positively by contributing to the abundance of shared mutualistic partner species (thus minimizing competition or the dilution of resources)^28,40^.

At the steady state, the network achieves a structure that optimizes the inherent tradeoffs. We examine the assembled structure under different intensities Ω_*m*_ and Ω_*c*_ of mutualism and competition. These parameters represent the interaction intensity per unit of niche proximity (see Methods). In this adaptive framework, changes in the interaction intensities can alter the network structure: the measures of nestedness NODF and modularity Q show a convex and concave dependence on the intensity of mutualism Ω_*m*_ (Fig. 2c, d), respectively. This corresponds to an adaptive linking strategy: modules tend to merge with enhanced mutualism (thus, lower modularity for larger module sizes and more intermodule links, and higher nestedness for more overlaps of partnership), but tend to split beyond a turning point Ω_*m*,*T*_ (see Supplementary Fig. 10a). These relations are generally robust regarding network size and aspect ratio of numbers of animals against plants (see sensitivity analyses in Supplementary Note 2).

On the other hand, enhancing the intensity of competition Ω_*c*_ contributes positively to the nestedness while suppresses the modularity (colored differently in Fig. 2c, d). This is due to competitive repulsion^26,27^: merging modules may counteract intensified competition by enlarging the separation between module hubs (Supplementary Fig. 10b). Thus, the so far underestimated within-guild competition actually acts as a crucial determinant for the cross-guild partnerships^23^.

### Transition in network stability

The adaptive niche model network reaches an asymptotically constant link structure and a balanced demographic distribution at the steady state (Supplementary Figs. 4c and 5a). It is useful to examine whether this population distribution on the evolved network can withstand transitory external interference. Here we characterize the stability of the assembled network by calculating the real part of the leading eigenvalue of the Jacobian of the population dynamics (Eq. (5)), *S* = −*Re*(*λ*)_*max*_ (Supplementary Note 3). This local stability measure considers the response to small perturbations on species abundances at a typical ecological timescale. In static theories, network stability calculations usually assume an fixed network topology under all external conditions^12,41–44^. An ecological network, however, typically changes its structure with the environment, resulting in changes in stability.

**Fig. 3:**
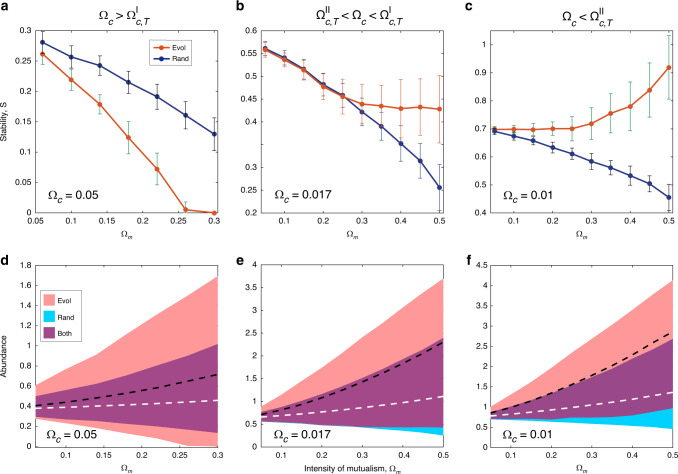
Stability of assembled network. **a**–**c** Transitions of stability induced by competition intensity. The relative stability is persistently negative or positive compared with the randomized network for the competition intensity Ω_*c*_ above or below the first threshold ${\Omega }_{c,T}^{I}\approx 0.025$ (between **a** and **b**). At the second threshold ${\Omega }_{c,T}^{II}\approx 0.015$, the regimes of competition intensity are separated where enhancing mutualism by Ω_*m*_ would stabilize or destabilize the network (between **b** and **c)**. The scenarios are shown for typical values at Ω_*c*_ = 0.05, 0.017, and 0.01 in the three intervals. The stability measures of the null model are shown by blue curves. Data are obtained from 50 simulation runs and presented as mean values ± SD. **d**–**f** Demographic distributions. The network stability is associated with the lower bound of the population of all species. The relative position of the population distributions of the assembled network (red area) to the random counterpart (blue area) changes with the competition intensity, which reverses the relative stability (compare **d** and **e**). On the other hand, enhancing mutualism is detrimental to the low-populated species at a relatively high competition level (**e**). Only at a sufficiently low competition level, the entire population and the stability rises with Ω_*m*_ (**f**). The average population per species 〈*n*〉 is marked by black and white dashed lines for the assembled and random network, respectively.

We examine the stability of the assembled network that has adapted to the interaction intensities (Fig. 2c, d). It shows both stabilizing and destabilizing roles of mutualism on the network stability. Most evidently, we identify two types of transitions in the stability induced by the overall intensity of competition at marked thresholds. The first threshold ${\Omega }_{c,T}^{I}$ differentiates the regimes of competition intensity, where the relative stability of the assembled network is persistently positive or negative compared with the randomized network (Fig. 3a, b). Hence, the network stability relative to random networks is predominantly subject to the competition level. The second threshold ${\Omega }_{c,T}^{II}$ separates the contrasting situations where enhancing mutualism by Ω_*m*_ would stabilize or destabilize the network (Fig. 3c). The comparative scenarios are shown for typical values of Ω_*c*_ in three intervals in Fig. 3a–c. The situations are blended in a narrow intermediate interval ${\Omega }_{c,T}^{II}\,<\,{\Omega }_{c}\,<\,{\Omega }_{c,T}^{I}$ where the network is more stable than the null model but enhancing mutualism still has a destabilizing role (Fig. 3b). Thus, whether enhancing mutualism stabilizes or destabilizes the evolved network is regulated by the competition intensity. Such bidirectional role of mutualism is persistently observed for different network sizes and aspect ratios (Supplementary Note 3).

The transitions are consistent with the change of demographic distribution. The network stability is related to the lower bound of the population of all species through their close relation $S=-Re({\lambda }_{m})\approx \min _{i}({n}_{i})$^21^. The relative stability of the evolved network as compared with its random counterpart can hence be considered as the relative position of their population distributions, which changes with the competition intensity. By intensifying the competition over the threshold ${\Omega }_{c,T}^{I}$, the lower bound of the population falls below that of the null model, which renders a negative relative stability (Fig. 3d, e). On the other hand, mutualism is prone to increasing the “rich” species but suppressing the “poor” at a high competition level. Only at a sufficiently weak competition level ${\Omega }_{c}\,<\,{\Omega }_{c,T}^{II}$ does enhancing mutualism boost the entire population, which contributes positively to the network stability (Fig. 3e, f). The bidirectional role of mutualism is exhibited on the stability mainly through the underpopulated species.

### Hysteresis in adaptation

**Fig. 4:**
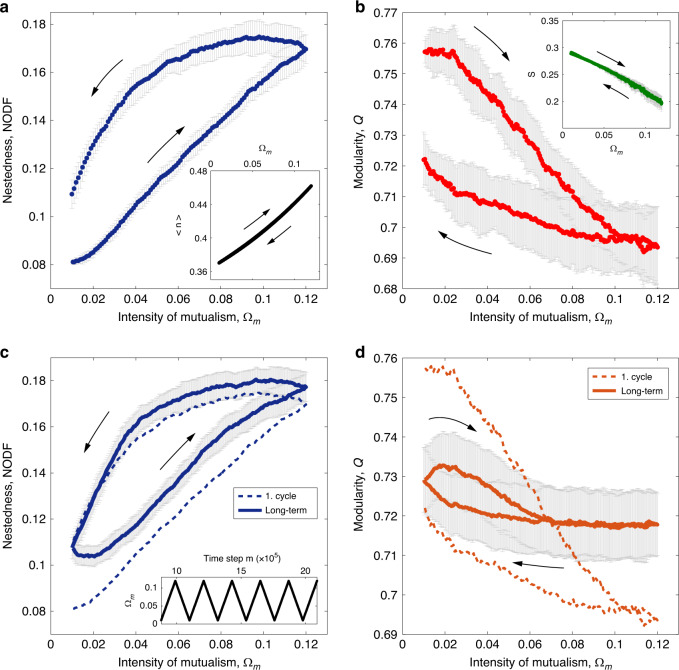
Asymmetric structural adaptation. **a**, **b** The nestedness and modularity measures show hysteretical trajectories with the intensity of mutualism Ω_*m*_, demonstrating asymmetric adaptation in opposite directions. A very slow change rate (*Δ*Ω_*m*_ = 10^−6^ per time interval) is used during the reciprocation of Ω_*m*_ so that the system is guaranteed to be always at quasi-steady-states. In contrast, the same population 〈*n*〉 (inset of upper panel) and stability *S* (inset of lower panel) are recovered on the altered network structure when Ω_*m*_ is restored. **c**, **d** Long-term response to cyclic change. The asymptotic behavior of the nestedness settles to a stable closed hysteresis loop. In contrast, the variation of modularity becomes narrowly constraint and more symmetric along both directions. Data are obtained from 50 simulation runs and presented as mean values ±  SD.

We further examine the resilience of the network structure under slow environmental changes. We found that such long-term changes may cause systematic alteration of the assembly that are irreversible even if the external environmental condition is restored^2,31,45^. Numerically, we track the structural measures of an assembled network by raising and restoring the mutualistic intensity Ω_*m*_, mimicking the impact of a gradually changing environment over a large time span. A slow change rate of Ω_*m*_(*t*) is used so that the entire process is guaranteed to be at quasi-steady-states. Strikingly, the trajectories of both nestedness and modularity show an unclosed hysteresis with the control parameter (Fig. 4a, b).

It shows an asymmetry in the adaptation: the process proceeds preferably in the direction of merging (when Ω_*m*_ increases) rather than splitting modules (when Ω_*m*_ decreases). Consequently, species engaged in cooperation may adopt alternative niche relations even under the same environmental condition. Such hysteresis implies that accidents of environmental history would be frozen in the mutualistic network structure^31,45^. (Such memory effect becomes less prominent for smaller-sized networks, see Supplementary Fig. 19).

However, this path-dependency affects only the structural properties. In the same process, the average community population 〈*n*〉 and the stability measure *S* are well recovered along the same path when the influenced variable Ω_*m*_ is restored (see insets of Fig. 4a, b). Hence, neither the overall population level nor local stability shows traces of environmental change, despite the drastically altered underlying structure.

The response to the long-term cyclic external change settles into a stable trajectory. The asymptotic behavior of the nestedness measure approaches a closed hysteresis loop with the continuously altering Ω_*m*_ after multiple cycles (Fig. 4c), remaining an asymmetry along the bidirectional paths. The change of modularity, in contrast, becomes more symmetric and narrowly constrained after the initial cycles (Fig. 4d), which infers the network has settled to a relatively robust modular structure.

### Invasion to mutualistic networks

At last, we demonstrate that the adaptive mechanism plays a crucial role in maintaining the patterns of mutualism during long-term evolution. We analyze the impact that invading mutant species have on the network structure. We adopt elements from the classical theory of “Adaptive Dynamics”^46–48^ and introduce an adaptive-evolutionary process based on two timescales: at large time intervals, mutant species, with a slightly different niche (trait) and a small proportion of the resident’s total abundance, attempt to invade a randomly chosen resident^49^. Between invasion attempts, the network undergoes a large number of short-timescale adaptive rewiring steps (see Methods and Supplementary Note 4) playing out whether the mutant survives, goes extinct, replaces, or coexists with the resident. Regardless, we show that a nontrivial nested and modular architecture persists in the presence of repeated invasions and extinctions.

**Fig. 5:**
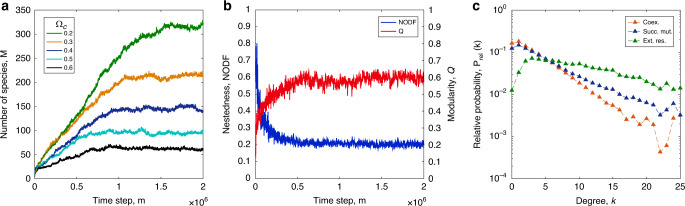
Invasions to mutualistic networks. **a** Starting from a small core of species (*M* = 4), the total number of species *M* increases and eventually saturates at a level that decreases with the competition intensity Ω_*c*_. **b** As *M* increases and stabilizes, the nestedness *NODF* decreases and modularity *Q* increases shown here explicitly for Ω_*c*_ = 0.4. **c** Starting from a large mutualistic network (*M* = 112) the relative probabilities for the survival of a mutant, the extinction of an invaded resident, and the coexistence of resident and mutant, as a function of resident degree *k*, measured *Δm* = 500 time steps after the invasion.

Starting from a small core of resident species, the number of existing species initially increases, saturating at a level referred to as the maximal capacity of the network, which decreases with the competition intensity (Fig. 5a). The nestedness initially decreases while modularity increases and both quickly saturate (Fig. 5b). Notably, the evolving structural measures show a distribution in the (*NODF*, *Q*) plane that overlaps with that of the empirical networks from the Web of Life data set (Supplementary Fig. 21b). Numerical simulations show that the invading mutants explore the entire niche space with wide-stretching phylogenetic trees rooted on the initial species (Supplementary Fig. 20).

The adaptive behavior is crucial for preserving nestedness. Without it, the nestedness would quickly disappear and the network size would grow in an unbounded way with repeated invasions (Supplementary Fig. 24). Adaptive rewiring leads to a strong degree-based bias in the mutants’ survival and removal chances due to extinction. Figure 5c shows the relative probabilities^50^ *P*_*rel*_(*k*) that the mutant has survived, that the mutant has coexisted with the resident, and that the invaded resident has gone extinct, examined at *Δm* = 500 time steps after the invasion. These probabilities show a clear tendency that generalists are more robust against invasions, whereas specialists are more likely to succumb (Supplementary Fig. 23). Thus, the cores of the modules are seldom affected by invasions and it is only specialists that are often substituted, which makes the nested and modular structure unaltered under repeated invasions.

## Discussion

We have developed an adaptive-network framework for the assembly of mutualistic networks, linking the structure of a network to the processes unfolding on it. By combining the concepts of niche structure and adaptive rewiring, the pervasively observed nested and modular patterns of mutualism emerge from an integrated underlying mechanism. Both patterns are complementary facets of optimal niche structure as was previously conjectured from heuristic arguments^51^. This integrated mechanism also provides an underlying explanation for the negative correlation between nestedness and modularity observed in empirical networks.

The proposed adaptive niche model extends the classical niche theory of Williams and Martinez^8,9^ (typically used for food webs) to a dynamical framework, using the original concept of niche interactions from MacArthur and Levin^26^. Although the classical niche theory is able to address the structure in a concise way, our model, at the cost of introducing more variables to describe niche-based interactions, can reveal a host of fundamental dynamical properties at different timescales. At the ecological timescale, the stability analysis demonstrates both stabilizing and destabilizing effects of mutualism, reconciling the contradictory conclusions of different theories^12,41–44^, in most of which the critical impact of competition has hitherto been largely ignored^23^. Counter-intuitively, it suggests that for a highly competitive community, enhancing mutualistic strength may detriment the low-fitness actors and reduce the network resilience, which is a side effect of cumulative advantage^38^. Furthermore, the striking hysteresis in structural adaptation suggests alternative stable structures can exist under the same external conditions^31,45^, implying that the traces of environmental history would be “frozen” in the ecological relationship or organizational partnership.

At the evolutionary timescale, the adaptive dynamics plays a crucial role in preserving network architecture under repeated invasions and extinctions. The invasion tests, based on a two-timescale adaptive-evolutionary dynamic process, suggest that successful surviving invaders can only enter the network at the “right” entry point, owing to the different resilience of resident nodes endowed by the architecture^46–48^. This may explain why the patterns of mutualism are persistently observed in nature in spite of repeated invasions and extinctions. Also, it suggests that the combination of processes at both timescales is an important scenario for network evolution^52^. Further results from the invasion tests show a tendency of trait convergence and complementarity (Supplementary Note 4). This is supported by strong evidence of selection favouring complementarity in mutualism^53^ and is consistent with theories assuming explicit coevolutionary mechanisms^54^.

Hence, this adaptive systems formulation has demonstrated a mechanism by which fundamental interactions can shape the global mutualistic network structure, providing a bottom–up perspective on mutualism. Indeed, from the network theoretical point of view, this mechanism has demonstrated that processes involving cumulative advantage or preferential attachment with local rules can be responsible not only for hierarchy formation but also for modularization^35–37^. In this manner, many general properties of mutualism that are nonspecific to constituents can be understood from the perspective of network dynamics. We anticipate that further investigations into questions about invasion and diversity^55–58^, cascading extinctions and resilience^59–64^, and restoration of mutualistic networks^65,66^ would be particularly revealing in this framework. The adaptive nature of niche relations should even underpin a broad category of interactions in socio-economic systems^4,67,68^.

## Methods

### Adaptive niche model

We consider a bipartite ecological network that contains interacting species of two guilds (denoted *P* and *A*, in analogy with plants and animal pollinators). We assign a pair of characteristics to each species: its niche and its abundance (individual species fitness). The niche profile is formulated as a Gaussian function *H*_*i*_(*s*) with uniform width *σ* and its center position $\bar{{s}_{i}}$ randomly chosen from the interval [0, 1] on a niche axis (Supplementary Note 1). Each species is involved in cross-guild mutualistic interactions with selected partners (represented by a matrix {*γ*_*ik*_}), in addition to competitive interactions with all rival species in its own guild ({*β*_*ij*_} for *A* or *P*). We define the niche proximity *H*_*ij*_ of a pair of interacting species *i* and *j* as

3 $${H}_{ij}=\int{H}_{i}(s){H}_{j}(s)ds=\exp \left(-\frac{{\left(\bar{{s}_{i}}-\bar{{s}_{j}}\right)}^{2}}{4{\sigma }^{2}}\right).$$

which refers to the total joint probability of possessing the same niche position for either competing species within the same guild^26,27^ or partner species across the guilds. The intensity of either type of pair-wise interaction is assumed to be proportional to the niche proximity *H*_*ij*_

4a $${\text{mutualistic}}\!\!:\,\quad {\gamma }_{{ik}}={\Omega }_{m}\cdot {\theta }_{{ik}}\cdot {H}_{ik}$$

4b $$\,{\text{competitive}}\!\!:\,\quad {\beta }_{ij}=\left\{\begin{array}{ll}1,&i=j\\ {\Omega }_{c}\cdot {H}_{ij},&i\,\ne\, j\end{array}\right.$$

where *i*, *j* ∈ *G* = (*A* or *P*) and $k\in \bar{G}=$(*P* or *A*). {*θ*_*ik*_} is the adjacency matrix, with the entries equal to 1 if *i* and *k* interact, and 0 if not. The mutualistic interaction strength is thus higher for partners located at similar positions across the guilds, corresponding to higher trait complementarity, whereas the competitive interaction strength is higher for rivals located at similar positions within the same guild, owing to higher trait similarity. The proportionality coefficients Ω_*m*_ and Ω_*c*_ are the interaction factors for mutualistic and competitive interactions, respectively, which capture the overall environmental influence.

The species abundances evolve according to a set of Lotka-Volterra equations with Holling Type II mutualistic functional response^1,12,21^:

5a $$\frac{d{n}_{i}^{A}}{dt}={n}_{i}^{A}\left({\rho }_{i}^{A}-\sum _{j}{\beta }_{ij}^{A}{n}_{j}^{A}+\frac{\sum _{k}{\gamma }_{ik}^{AP}{n}_{k}^{P}}{1+h\sum _{k}{\theta }_{ik}^{AP}{n}_{k}^{P}}\right)$$

5b $$\frac{d{n}_{i}^{P}}{dt}={n}_{i}^{P}\left({\rho }_{i}^{P}-\sum _{j}{\beta }_{ij}^{P}{n}_{j}^{P}+\frac{\sum _{k}{\gamma }_{ik}^{PA}{n}_{k}^{A}}{1+h\sum _{k}{\theta }_{ik}^{PA}{n}_{k}^{A}}\right)$$

where the coupling strengths {*γ*_*ik*_} and {*β*_*ij*_} are defined above. Note that *k* actually spans only species interacting with *i*.

Rewiring and update of niche proximity: all species are assigned uniform abundances *n*_*i*_ = *n*_0_ and connected to partner species across the guilds uniformly at random with a specified connectance *C*_0_ in the initial condition. The system evolves according to Eq. (5) and at each time interval *t* = *mT* (*m* is a positive integer), a species *i* is chosen uniformly at random and one of its existing links *γ*_*ij*_ is rewired to a randomly selected different mutualistic partner species *k* with probability *p*_*ij*_. The niche proximity across the guilds and the mutualistic coupling strength are updated: *H*_*ij*_ → *H*_*ik*_ and *γ*_*ij*_ → *γ*_*ik*_. *T* is chosen to be sufficiently large to guarantee that the population dynamics reaches equilibrium between subsequent rewiring attempts. At the end of the time interval $t^{\prime} =(m+1)T$, the abundance of species *i* is compared with the previous value^20,21^. If ${n}_{i}(t^{\prime} )\,> \,{n}_{i}(t)$, the rewiring is accepted; otherwise the previous link *ij* is restored and the niche proximity is recovered. The rewiring probability for the link *ij* is ${p}_{ij}=1-{k}_{j}^{-\eta }$ (*η* > 0), with *k*_*j*_ being the degree of the partner species, so that a species with a lower degree is prone to keeping its link(s). This guarantees that any species interacts with at least one mutualistic partner species, so that the connectance of all interacting species remains constant during rewiring.

We use random networks as the null model, where the probability of each entry of the adjacency matrix being occupied is the average of the occupation probabilities of the row and column^6^.

### Invasion

We use a two-timescale process: at large time intervals (*T*_*m*_ = *R*_*T*_ $*$ *T*), a resident species is randomly chosen with a probability proportional to its abundance *n*_*i*_ and a mutant species is created which inherits all the mutualistic links and a small proportion of the resident’s total abundance (1%) (Supplementary Note 4). The mutant deviates from the resident by a displacement on the niche axis, drawn from a normal distribution. Extra links are randomly deleted to stay in accords with the empirical relation between connectance and network size *C*_0_ = 4/*M*^0.8^. At short time intervals (*T*), adaptive rewiring, as detailed above, occurs. Invasions to two types of initial networks are analyzed: (1) a small core of resident species, consisting of two pairs of species (one in each guild) with all cross-guild connections; (2) an assembled network of a relatively large size (112 species).

### Reporting summary

Further information on research design is available in the Nature Research Reporting Summary linked to this article.

## Supplementary information

Supplementary Information

Reporting Summary

## Data Availability

The Web of Life data set used in this study is available at http://www.web-of-life.es/.

## Code availability

The codes for simulating the core model are available at: https://github.com/tsaiwr/adaptive-niche-model (10.5281/zenodo.4033975).
