# Supplementary material for: Mutualistic networks emerging from adaptive niche-based interactions
Source: Nat Commun. 2020 Oct 29;11:5470. doi: 10.1038/s41467-020-19154-5 (PMC7596068; doi:10.1038/s41467-020-19154-5)
Supplement: Supplementary file 1 — Supplementary Information [file 41467_2020_19154_MOESM1_ESM.pdf]

Supplementary Information
Mutualistic Networks Emerging from Adaptive Niche-Based Interactions

W. Cai, J. Snyder, A. Hastings and R. M. D'Souza

We present further structural and dynamical properties of the mutualistic network based on the adaptive niche model. The contents are organized as follows. Detailed definition of the model and numerical implementation are provided in the first Supplementary Note 1. We address the structural properties beyond those discussed in the main text in Supplementary Note 2, covering sensitivity of parameterization and the structure-population relation. Further dynamical properties are discussed in Supplementary Note 3 and 4, which are dedicated respectively to the network resilience at the ecological timescale and robustness of network structure under invasions at the evolutionary timescale. In Supplementary Note 5, we discuss the noise effect that leads to sub-optimal network structures.

SUPPLEMENTARY NOTE 1: ADAPTIVE NICHE MODEL (DEFINITION AND SIMULATION)

We aim at generating a structured network from iterative local interactions in a low-dimensional niche space. In general, an ecological system may involve multiple types of signed interactions. For a mutualistic system specifically, both mutualistic interactions $(+,+)$ across the guilds and competitive interactions $(-,-)$ within guilds need to be taken into consideration. From the perspective of Hutchinsonian niches, competing actors with their intrinsic niche profiles interact adaptively in order to maximize the individual node fitness in a community [1]. This concept can be generalized to a networked system with more than one type of interactions, resulting in an optimal niche relation and demographic distribution.

Model definition. We consider an ecological mutualistic network consisted of M_P plant species and M_A animal pollinator species in two guilds, respectively. The intrinsic living condition of each guild of species is represented by a one-dimensional niche axis. Each of the $M = M_P + M_A$ species is assigned a pair of characteristics, a niche function $H_i(s)$ and an abundance n_i as its fitness. The niche profile is characterized by a Gaussian function

$$H_i(s) = \frac{1}{\sqrt{2\pi}\sigma} e^{-(s-\overline{s_i})^2/2\sigma^2} \tag{1}$$

which is interpreted as the probability density of occupying the position s on the niche axis [2]. The niche centers $\overline{s_i}$, as the mean niche positions, are uniformly dispersed on the niche axis $[0,1]$ at random. We adopt a uniform niche width σ for simplicity.

Species are involved in the cross-guild mutualistic interactions with selected partner species, while compete with all rival species within its own guild. For either type of interaction, we define the niche proximity H_{ij} for a pair of interacting species as

$$H_{ij} = \int H_i(s) H_j(s) ds = e^{-(\overline{s_i}-\overline{s_j})^2/4\sigma^2}. \tag{2}$$

For within-guild competition, the niche proximity corresponds to the trait similarity of rival species (animal-animal or plant-plant), while for cross-guild mutualism, it refers to the trait complementarity of the partner species (animal-plant). The niche proximity can then be interpreted as the total joint probability of occupying the same position in the same position on the same niche axis or in matching positions on two niche axes.

We represent the coupling relation by the matrix γ^{AP} for mutualistic relations and β^A or β^P for competitive relations in respective guild A or P. For either type of interaction, we assume that the coupling strength is proportional to the niche proximity of two species

$$\text{mutualistic:} \quad \gamma_{ik} = \Omega_m \cdot \theta_{ik} \cdot H_{ik} \tag{3a}$$

$$\text{competitive:} \quad \beta_{ij} = \begin{cases} 1, & i = j \\ \Omega_c \cdot H_{ij}, & i \neq j \end{cases} \tag{3b}$$

where $i, j \in G = A$ or P and $k \in \bar{G} = P$ or A. The coefficients of proportionality Ω_m and Ω_c are the interaction intensities for mutualistic and competitive interactions, respectively, which represent the interaction strength per unit of niche proximity measure. $\{\theta_{ik}\}$ is the adjacency matrix: $\theta_{ik} = 1$ if species i and k interact, and 0 if not.

In the initial state, a uniform abundance is assigned to all M species $n_i = n_0$ at $t = 0$. For cross-guild mutualistic interactions, species are randomly connected across the guilds with a connectance C_0. Concretely, for each pair of species i and j ($i \in [1, M_A]$, $j \in [1, M_P]$), a random number r is generated uniformly in $[0,1]$. A connection is formed if $r < C_0$ and left absent otherwise. We consider a process of niche-based adaptive connection at the ecological timescale. At constant time intervals, species rewire repeatedly to change their niche relations and proximity in attempt to maximize the individual node fitness [3, 4]. The following updating rules are given.

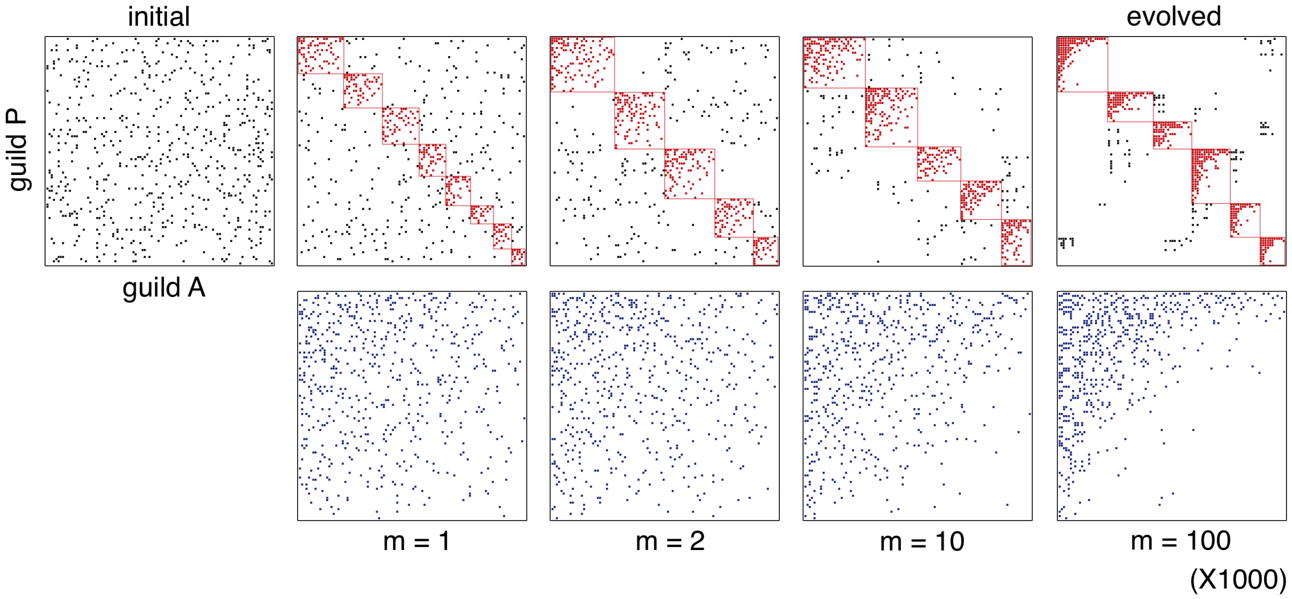

Supplementary Figure 1. Emergence of network patterns. Starting from an initial random bipartite network of $M_A = 100$ animal species and $M_P = 100$ plant species, an architecture of both modular (upper panel) and nested (lower panel) patterns is established through the adaptation of niche relations. The snapshots of temporal link patterns are illustrated by the adjacency matrices at different times ($m = 1000, 2000, 10000$ and 10^5). The matrices are sorted by the modules and then by degrees within them for showing the modular structure, and simply by degrees for showing the nested structure, respectively. We mark the entries within the partitioned modules in red and those lying outside in black. The links are gradually absorbed into the modules (blocks in the upper panels) as the species are attaching to the local generalist hubs (upper left corners of the blocks). The example network, simulated for $C_0 = 0.058$ and $(\Omega_m, \Omega_c) = (0.10, 0.05)$, is significantly modular and nested ($Q = 0.6207$ and $NODF = 0.1666$) at the steady state, compared with randomized networks ($p < 0.0001$; two-sided t-test).

I. Rewiring and changing niche proximity: At the beginning of each time interval $t = mT$ (m is a positive integer), we randomly select a species i and one of its existing links γ_{ij} is rewired to a randomly selected different mutualistic partner species j' with probability p_{ij}: $\gamma_{ij} \longrightarrow \gamma_{ij'}$. When a new partner is connected, the mutualistic coupling strength is changed according to the niche proximity of the new species pair, that is, $\gamma_{ij'} = \Omega_m \cdot H_{ij'}$ (Supplementary Eq. 3a), and set $\gamma_{ij} = 0$.

The rewiring probability is set to be $p_{ij} = 1 - k_j^{-\eta}$ ($\eta > 0$). As such, species with a large number of partners are tolerant in losing links while species with a small number of partners tend to keep them. Note that by using this condition, all participating species are guaranteed to have at least one partner, so that the connectance (or total number of links) of interacting species is constant over time. The competitive coupling strengths $\{\beta_{ij}\}$ do not change over time, since we assume here fixed niche positions at the ecological timescale (also see the main text and Supplementary Note 4 for the case of changeable niches at the evolutionary timescale).

II. Population dynamics: After the rewiring and update of niche proximity, we allow the abundances of all species to settle to a new equilibrium, according to the generalized Lotka-Volterra equations with Holling-Type II mutualistic functional response [5, 6]

$$\frac{dn_i^A}{dt} = n_i^A \left(\rho_i^A - \sum_j \beta_{ij}^A n_j^A + \frac{\sum_k \gamma_{ik}^{AP} n_k^P}{1 + h \sum_k \theta_{ik}^{AP} n_k^P} \right) \tag{4a}$$

$$\frac{dn_i^P}{dt} = n_i^P \left(\rho_i^P - \sum_j \beta_{ij}^P n_j^P + \frac{\sum_k \gamma_{ik}^{PA} n_k^A}{1 + h \sum_k \theta_{ik}^{PA} n_k^A} \right) \tag{4b}$$

where ρ_i is the intrinsic growth rate, $\{\gamma_{ij}\}$ and $\{\beta_{ij}\}$ are coupling strengths proportional to the niche proximity values (Eq. 3), and h is the handling time. The time interval T is set sufficiently large to guarantee the dynamics to reach an equilibrium.

III. Link recovery. At the end of the time interval $t' = (m + 1)T$, we compare the current abundance of species i to the previous value. If $n_i(t') > n_i(t)$, we keep the new link $\gamma_{ij'}$ as is; otherwise, we recover the link $\gamma_{ij'} \longrightarrow \gamma_{ij}$ and

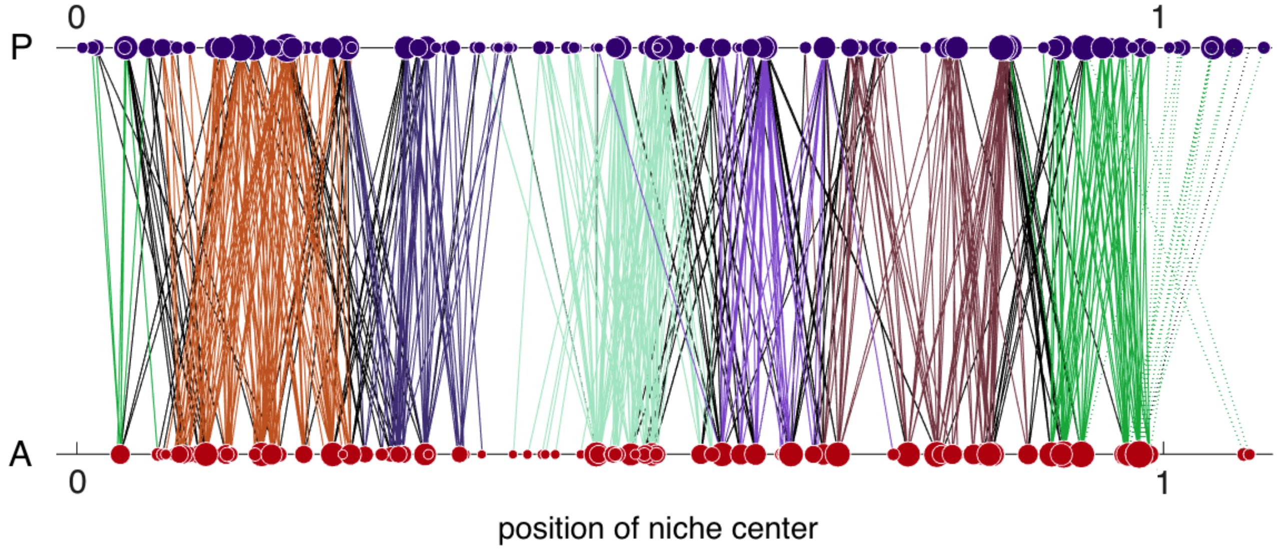

Supplementary Figure 2. Link pattern and distribution of abundances at steady state. Links belonging to different modules are marked in colours, according to the partition of the final snapshot in Supplementary Fig. 1. The modules are connected by the black-coloured links lying outside all modules. The dotted lines are links that wind around the periodic boundary (nodes close to the right side of $s = 0$ are duplicated at $s > 1$ for display). The species abundances, as represented by the sizes of the disks, show a heterogeneous demographic distribution along the niche axis.

the niche proximity $H_{ij'} \longrightarrow H_{ij}$.

Numerical simulation. The initial random network develops into both highly modular and nested patterns by following the above process, as shown in Supplementary Fig. 1 and 2. All macroscopic structural and demographic measures settle to constants asymptotically at the steady state (Supplementary Fig. 4c and 5a). Most rewiring attempts are rejected at the steady state, which keep fluctuations of these measures within a narrow range.

We use the following settings for numerical simulations. The connectance is chosen to be $C_0 = 4/M^{0.8}$, which is drawn from the fitting for empirical networks [4] (see Supplementary Fig. 3b). Specifically, we show the results for $M_A = M_P = 100$ and $C_0 = 0.058$ in the main text and this supplementary information, unless specified otherwise. We set the exponent $\eta = 1$ in the rewiring probability; we find that simulation results are insensitive to the choice of η in a large range ($\eta \in [1, +\infty)$). A uniform intrinsic growth rate $\rho = 1$ and niche width are set for all species ($\sigma = 0.1$ is used for Fig. 1, 2 (except 2a), 3 and 4 in the main text). The handling time h determines the saturation level in the functional response [6], which is set to 0.1. We use randomized networks as the null model, where the probability of each entry of the adjacency matrix being occupied is the average of the occupation probabilities of the row and column [7].

To avoid boundary effects, we define the niche axis to be periodic so that species are roughly symmetric on the niche axis: each species has approximately equal numbers of rival species in the same guild and potential mutualistic partner species in the opposite guild. Thus the pairwise niche proximity is always calculated for the shortest niche distance $\|\overline{s_i} - \overline{s_j}\|$ in Supplementary Eq. 2. If a fixed niche boundary condition is used, the simulation results are qualitatively similar. The niche centers are still confined within the interval $[0, 1]$, except that the niche proximity should be calculated on the one-dimensional axis without periodic condition [8].

The nestedness is measured by the metric $NODF$ (nestedness metric based on overlap and decreasing fill) [9]. The modularity of the adjacency matrix θ_{ij} is calculated by seeking a partition of the network that maximizes the modularity quality function

$$Q = \frac{1}{2L} \sum_{i \in A, j \in P} \left(\theta_{ij} - \frac{k_i k_j}{2L} \right) \delta(c_i, c_j) \tag{5}$$

where k_i is the node degree, $2L$ is the total number of links, and c_i is the module that node i belongs to under a certain partition. For any snapshot of the adapting network, we use the leading eigenvector algorithm [10] to obtain the modularity value. We used the tool package BiMat for calculating these structural measures [11, 12].

For the comparison with empirical networks, we calculate the dyadic structural measures ($NODF$, Q) of an ensemble of 300 generated networks: We set the exterior characteristics for each network according to empirical networks

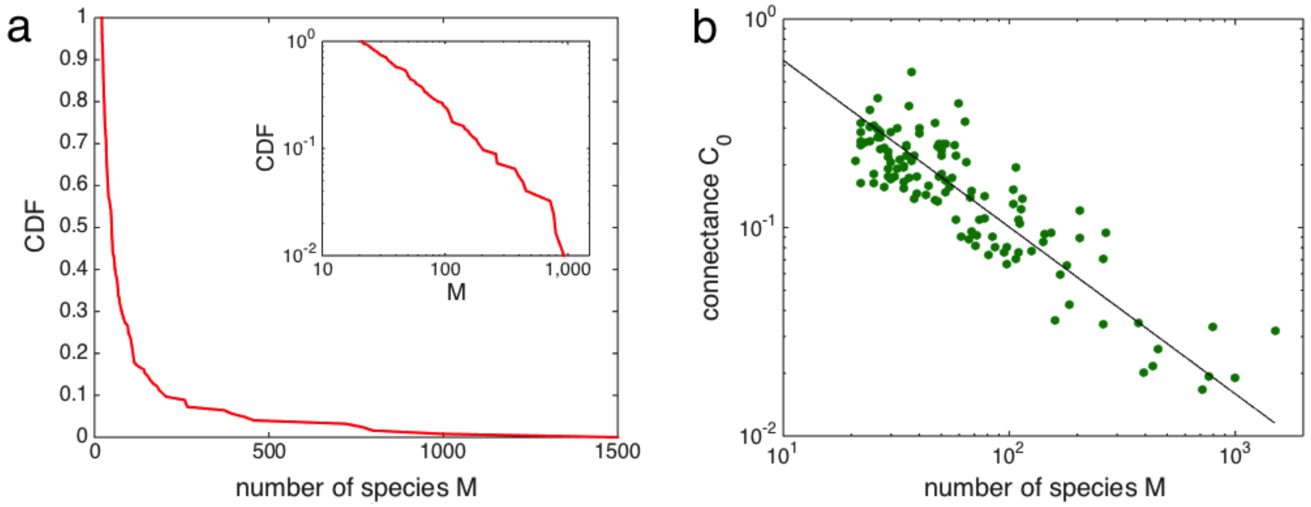

Supplementary Figure 3. Measures of empirical ecological networks from the *Web of Life* [13]. The dataset contains 144 ecological mutualistic networks of different geographical factors and constituents. The size of these empirical networks M ranges from 21 to 1500, with an average of 112, which follows a fat-tail distribution (**a**), while the connectance C_0 is approximately related to M by $C_0 = 4/M^{0.8}$ (**b**, linear fitting in the log-log scale). The aspect ratio of animal and plant species $R_M = max(M_A/M_P, M_P/M_A)$ ranges from 1 to 9.8, with an average value of 2.9.

while choose interaction intensities and niche width randomly. In concrete, we set M_A, M_P and C_0 according to a randomly chosen network from the *Web of Life* dataset, which contains 144 ecological mutualistic networks of different geographical factors and constituents [13]. The size of these empirical networks M ranges from 21 to 1500, with an average of 112, which follows a fat-tail distribution (Supplementary Fig. 3a), while the connectance C_0 is approximately related to M by $C_0 = 4/M^{0.8}$ (Supplementary Fig. 3b) [4]. The aspect ratio of animal and plant species $R_M = max(M_A/M_P, M_P/M_A)$ ranges from 1 to 9.8, with an average value of 2.9. On the other hand, we choose interaction intensities (Ω_m, Ω_c) uniformly from $[0.01, 0.30] \times [0.01, 0.30]$ and niche width from $[0.01, 0.50]$ at random. Data points are only shown for networks at feasible equilibria. The comparison of the two bands of points ($NODF$, Q) is shown in Fig. 2a in the main text. The negative correlations are fitted linearly for both bands.

Time course of adaptation. We show the time course of the adaptation of an example network ($M_A = M_P = 100$) in Supplementary Fig. 4. By using the multislice partitioning algorithm GenLouvain [14, 15] (for robust partitioning of temporal networks), one observes that the modules reduce in the number rapidly in the transitory stage (first several thousands of time steps, Supplementary Fig. 4a) and the network stabilizes to a robust partition at the steady state (Supplementary Fig. 4b), where both structural measures ($NODF$ and Q) reach a flat plateau ($m > 10^5$ in Supplementary Fig. 4c). The stationarity of a module can be characterized by the Jaccard index, which compares the contents of the successive modules that are separated by Δm time steps

$$J_\alpha(m, m - \Delta m) = \frac{|A_\alpha(m) \cap A_\alpha(m - \Delta m)|}{|A_\alpha(m) \cup A_\alpha(m - \Delta m)|} \tag{6}$$

where A_α denotes the members of a specific module in focus [15, 16]. Thus, the Jaccard index describes the similarity of successive modules, ranging from 0 (no common member) to 1 (identical). The index is significantly lower than 1 in the transitory period, while it approaches a value close to 1 at the steady state, showing a highly robust modular structure (Supplementary Fig. 4d).

Along with the adaptation of the structure, the demographic distribution of the species also reaches a steady state (Supplementary Fig. 5a). The resilience of this distribution under perturbation is characterized by the linear stability measure S (see Supplementary Note 3), which is bounded within a small fluctuation around a fixed value (Supplementary Fig. 5b).

The adaptation process proceeds through a positive feedback of cumulative advantages, linking the structural and demographic variables: a species with more partners tends to gain a higher abundance, which in turn attracts even more partners. This is reflected in the growing positive correlation of the normalized abundance $\hat{n}_i = (n_i - \langle n \rangle)/\sigma(n)$ and normalized degree $\hat{k}_i = (k_i - \langle k \rangle)/\sigma(k)$ (Supplementary Fig. 5c). The two normalized quantities characterize the demographic and structural advantage of a species compared with other species, respectively, where the symbol $\langle \dots \rangle$ and $\sigma(\dots)$ denote the mean and standard deviation over all species. The average p-value $\bar{p} < 0.0001$ (two-sided

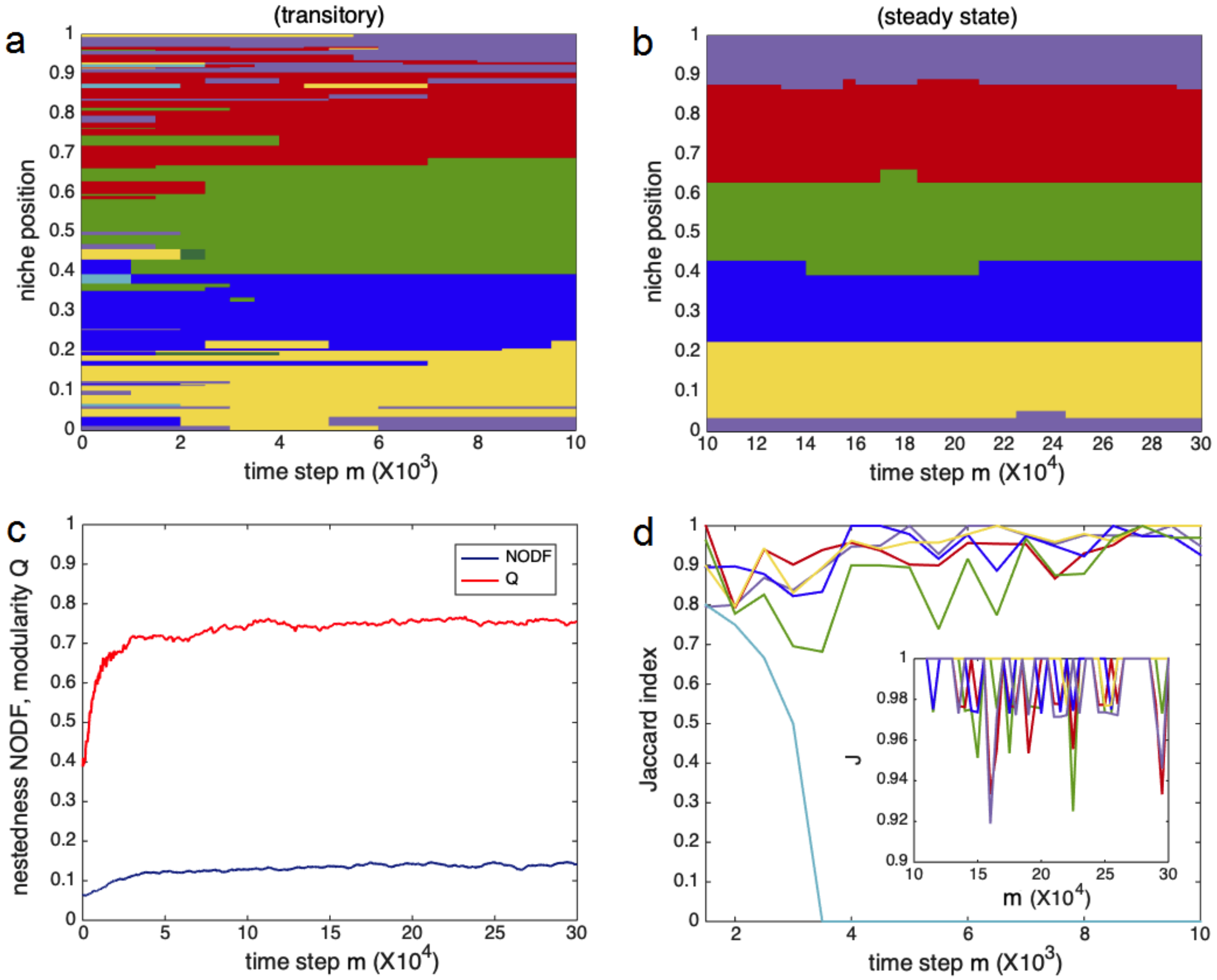

Supplementary Figure 4. Time course of adaptation. **a, b**, Temporally changing network partition. Each colour represents a module, in which species are more densely connected internally than to those in other modules. The number of modules decreases rapidly in the transitory period (a) and stabilizes at the steady state (b). The temporal partitioning is obtained at a resolution of 500 time steps in the transitory period (a) and 5000 at the steady state (b), respectively (the partitions are shown for guild A). **c**, Structural measures. Both modularity Q and nestedness $NODF$ of the example network reach relatively constant values after a transitory time period. **d**, Robustness of modules. The Jaccard index describes the similarity of successive modules, ranging from 0 to 1. The main plot shows that the index is significantly lower than 1 in the transitory period, where the modules are compared every $\Delta m = 500$ time steps. The index approaches a value close to 1 at the steady state (inset), showing a highly robust modular structure, where the modules are compared every $\Delta m = 5000$ time steps. $M_A = 100$ animal species and $M_P = 100$ plant species are involved in the simulations. The example network is simulated with $\Omega_m = 0.05$, $\Omega_c = 0.10$, $\sigma = 0.1$ and $C_0 = 0.058$.

t-test) is calculated for the last 10^5 time steps. The positive correlation of $\hat{n}_i$ and $\hat{k}_i$ of the network at steady state is explicitly shown for a snapshot in the inset of Supplementary Fig. 5c.

Moreover, the structural advantage of an individual is also correlated with the broadness of its partners. We define the broadness of partners of a given species i as $r_i = \sigma_j(d_{ij})$, where $d_{ij} = \|\bar{s}_i - \bar{s}_j\|$ is the niche distance between species i and its partner j, $\sigma_j(\dots)$ is the standard deviation over all partners of species i. The normalized broadness $\hat{r}_i = (r_i - \langle r \rangle)/\sigma(r)$ and the normalized degree $\hat{k}_i$ is again positively correlated (but more weakly, as seen from the average p-value $\bar{p} = 0.0026$) (Supplementary Fig. 5d). This reflects that a species with more partners tends to have partners that spread over a broader diversity of traits. These two aspects clearly realize positive feedbacks among the advantage in the abundance, that in the degree (how many partners one has) and that in the broadness of partnership (how diverse the partners are) .

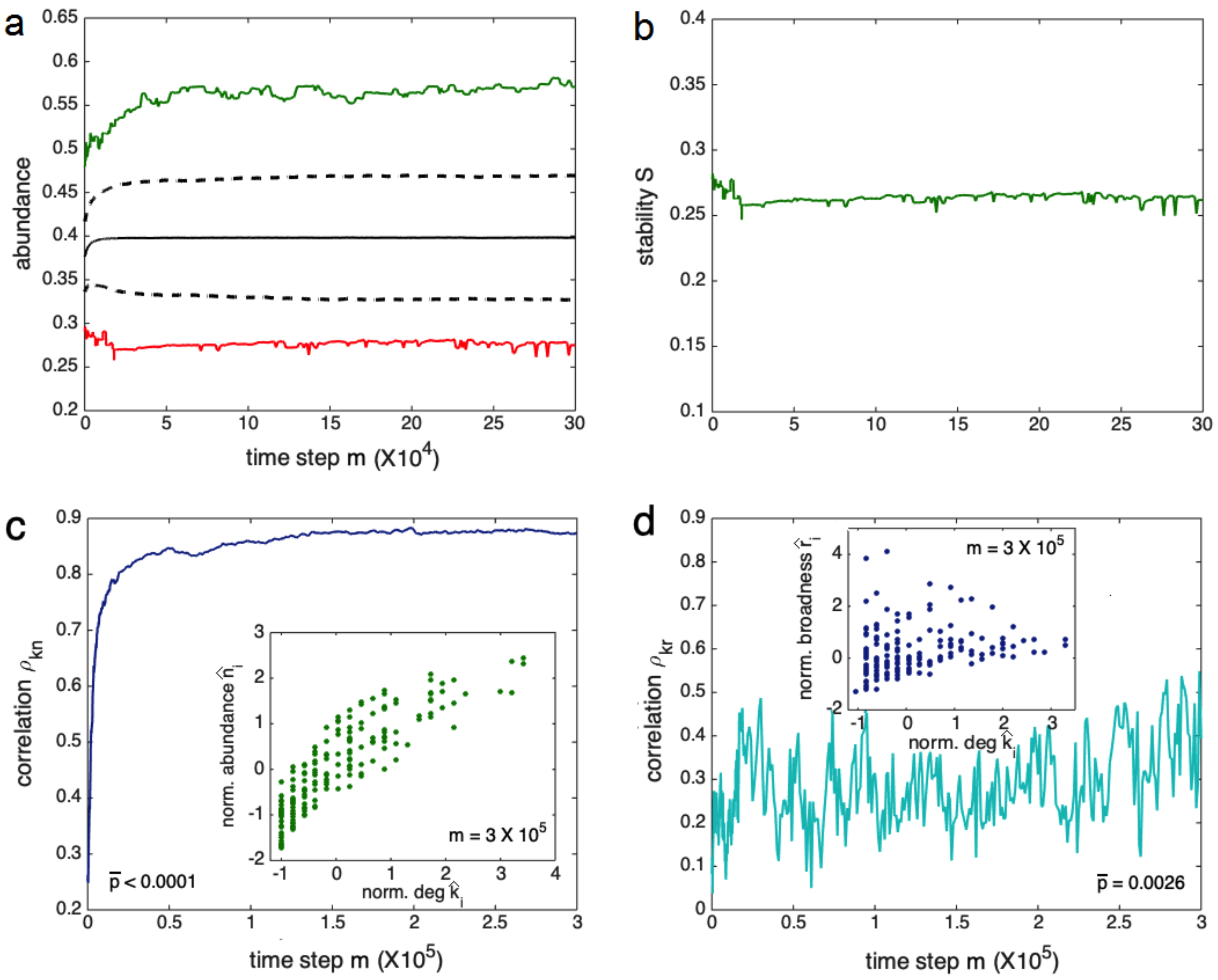

Supplementary Figure 5. Population and stability in the adaptive process. **a**, Time courses of population of the example network in Supplementary Fig. 4, showing the mean abundance per species $\langle n \rangle$ (solid black), the deviation $\sigma(n)$ over the community (dashed black), and the lower and upper bounds n_{min} and n_{max} (solid red and green). **b**, Time course of local stability. The network stability $S = -Re(\lambda)_{max}$ is measured by the real part of the leading eigenvalue of the Jacobian matrix of Supplementary Eq. 4 at the end of every time interval, when the population have settled to an equilibrium. **c**, The correlation (Pearson's coefficient) of normalized individual species abundance $\hat{n}_i$ and normalized degree $\hat{k}_i$ becomes highly correlated at the steady state (shown for one simulation run). The average p-value $\bar{p} < 0.0001$ (two-sided t-test) is calculated over the last 10^5 time steps. Inset: Positive correlation between $\hat{n}_i$ and $\hat{k}_i$ for a snapshot of the network at steady state (at the time step $m = 3 \times 10^5$). **d,** The normalized broadness of partnership $\hat{r}_i$ and normalized degree $\hat{k}_i$ becomes correlated (measured by Pearson's coefficient) in the adaptation. The average p-value $\bar{p} = 0.0026$ (two-sided t-test) is calculated over the last 10^5 time steps. Inset: Positive correlation between $\hat{r}_i$ and $\hat{k}_i$ for a snapshot at $m = 3 \times 10^5$.

SUPPLEMENTARY NOTE 2: STRUCTURAL PROPERTIES AT STEADY STATE

Numerical simulations show that all macroscopic structural and demographic measures are asymptotically constant at the steady state. Here we further address the intrinsic relations of these measures that have been established in the adaptive process.

Scaling and aspect-ratio effects on structure. We have shown results for relatively large-sized networks ($M = 200$) for general interest in the main text, because a statistical system is typically subject to strong stochastic fluctuations at small sizes while such fluctuations diminish by approaching a large-size limit (claim of central limit theorem). On the other hand, although only 11% of ecological mutualist networks from the Web of Life Dataset contain more than 200 species (Supplementary Fig. 3), an industrial mutualistic network may contain several thousands of actors (e.g., designer-contractor partnership in garment industry [17]).

The system size and aspect ratio however play an important role in determining the network structure. We examine the scaling of network structure with the total number of species M (network size) while fix the aspect ratio $R_M = 3$ (see Supplementary Fig. 6a and 6b). With the interaction intensities and niche width fixed, the nestedness $NODF$ and modularity Q reach relatively constant values at large sizes (simulated with the relation $C_0 = 4/M^{0.8}$). For smaller sizes, the finite size effect is significant by showing a large deviation in the structural measures from those for large system sizes. On the other hand, the nestedness $NODF$ and modularity Q show a linear dependence on the

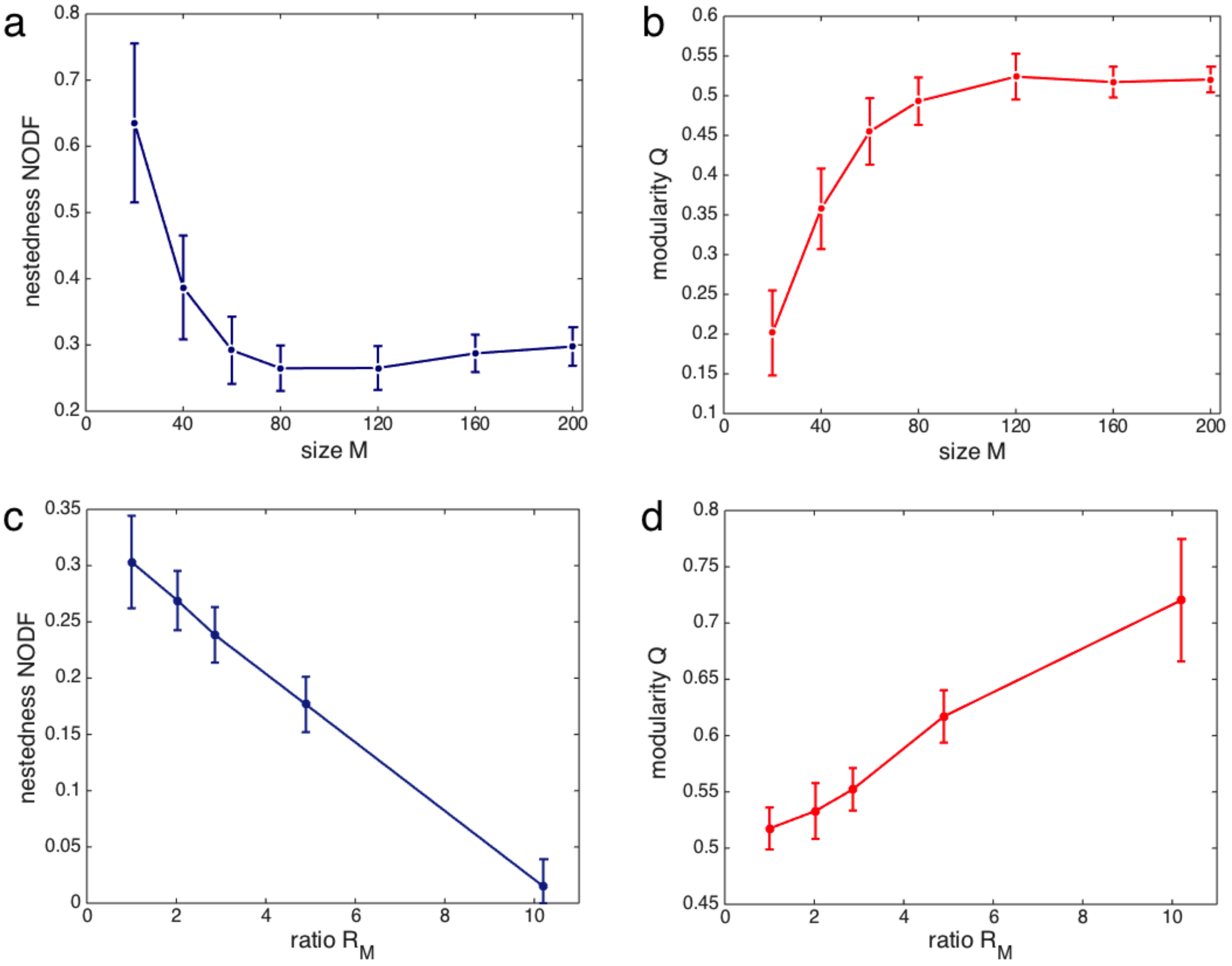

Supplementary Figure 6. Dependence of nestedness $NODF$ and modularity Q on the number of species M and the aspect ratio $R_M = M_A/M_P$. R_M is fixed at 3 in (**a, b**). M is fixed at 112 in (**c, d**). The parameters are chosen as follows: $\Omega_m = 0.05$, $\Omega_c = 0.05$ and $\sigma = 0.2$. Data are obtained from 25 simulation runs and presented as mean values ± SD.

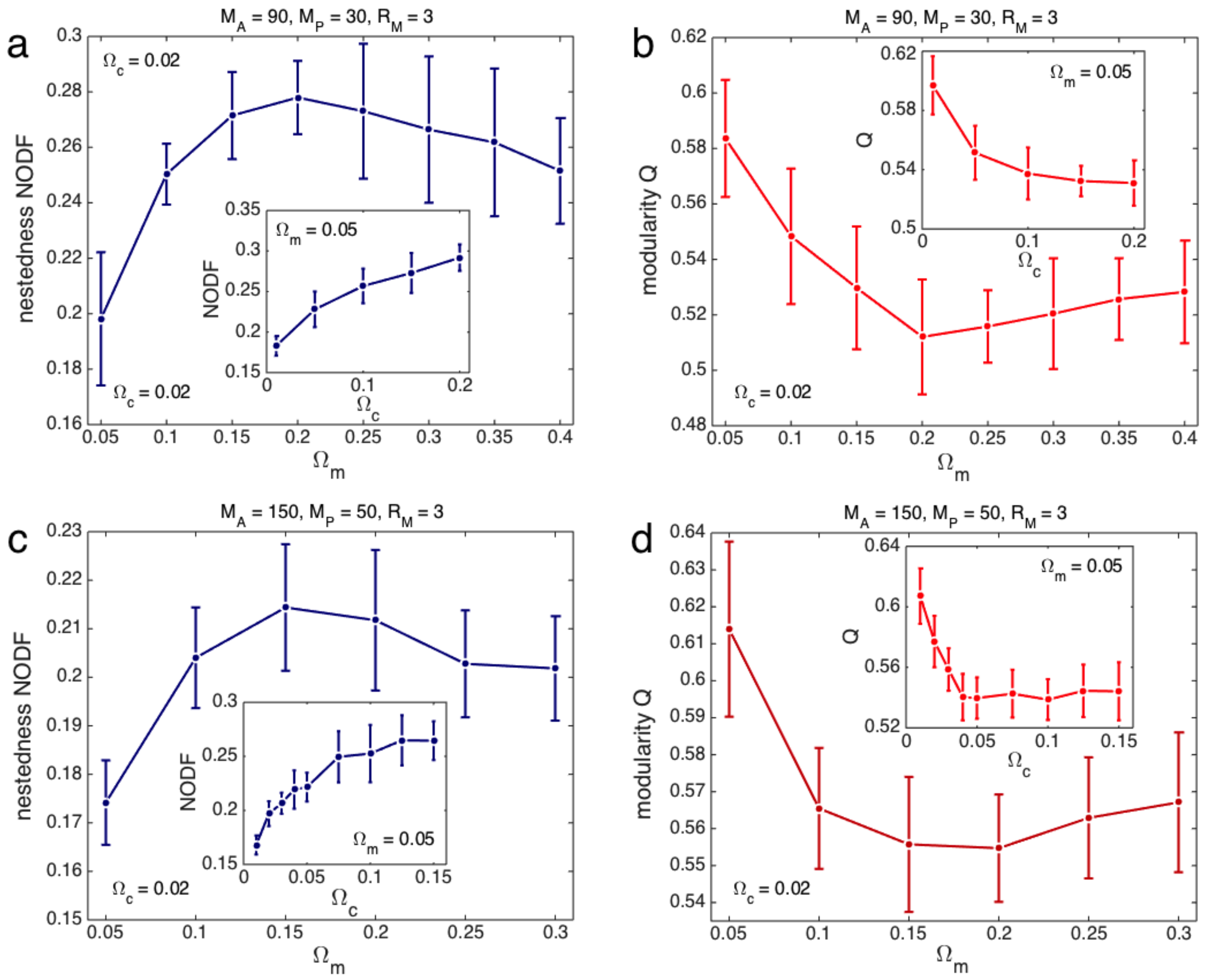

Supplementary Figure 7. Structural measures versus interaction intensities for different network sizes $M = M_A + M_P$. The nestedness $NODF$ and modularity Q have a convex and concave dependence on the mutualistic intensity Ω_m, respectively. $NODF$ and Q are monotonically dependent on Ω_c. The total number of species M is chosen to be 120 (**a, b**) and 200 (**c, d**), repectively, while the aspect ratio is fixed $R_M = M_A/M_P = 3$. The niche width is chosen to be $\sigma = 0.2$. Data are obtained from 25 simulation runs and presented as mean values ± SD.

aspect ratio R_M, which ranges in $[1, 10]$ with the total number of species fixed at $M = 112$.

However, the dependence of network structure on the interaction intensities is qualitatively unchanged with either M or R_M. To demonstrate this, we examine the relations of $NODF$ and Q with Ω_m and Ω_c by using the following settings: (1) We set two different system sizes M at 120 and 200 with a fixed $R_M = 3$, as shown in Supplementary Fig. 7; and (2) we set two different aspect ratios R_M at 1.0 and 2.9 (average of empirical networks) with a fixed $M = 112$ (average of empirical networks), as shown in Supplementary Fig. 8. For all these combinations, we observe the general tendencies: the nestedness $NODF$ and modularity Q have a convex and concave dependence on the mutualistic intensity Ω_m, respectively, while they have a monotonic relation with the competitive intensity Ω_c (insets). Such tendencies are in accordance with those shown in Fig. 2c and 2d of the main text.

A necessary remark should be made on the relation between network structure and connectance. Despite that we have used the fitted relation between the size M and connectance C_0 in the above analyses, one would observe a limited change in the nestedness and modularity if C_0 is still allowed to change within a small vicinity of $\langle C_0 \rangle = 4/M^{0.8}$. We have simulated such small changes of C_0 with a fixed size M. As shown in Supplementary Fig. 9, the nestedness and modularity exhibit a positive and negative dependence on the connectance, respectively. The tendency is rendered simply due to the propensity that addition of links contributes to higher probability of overlaps of partnership and more inter-module links.

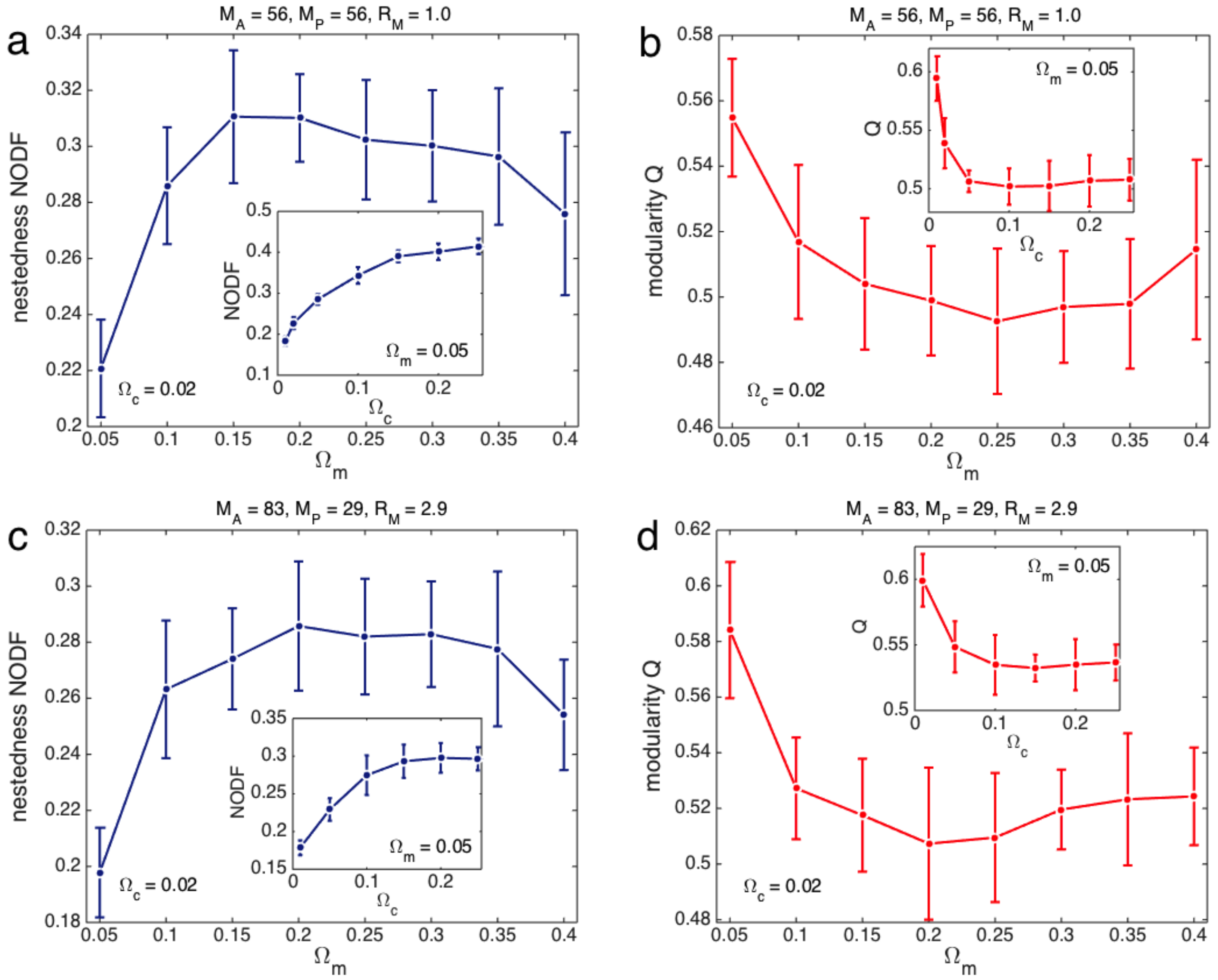

Supplementary Figure 8. Structural measures versus interaction intensities for different aspect ratios $R_M = M_A/M_P$. The nestedness $NODF$ and modularity Q have a convex and concave dependence on the mutualistic intensity Ω_m, respectively. $NODF$ and Q are monotonically dependent on Ω_c. The ratio R_M is chosen to be 1.0 (**a, b**) and 2.9 (**c, d**), repectively, while the total number of species is fixed $M = M_A + M_P = 112$. The niche width is chosen to be $\sigma = 0.2$. Data are obtained from 25 simulation runs and presented as mean values ± SD.

Merging and splitting of modules. Changes in the interaction intensities regulate the assembly of network through merging or splitting of modules. As shown by the number of modules N_m in Supplementary Fig. 10a, modules tend to merge with enhanced mutualism before reaching a turning point $\Omega_{m,T}$, which leads to lower modularity for increased module sizes and intermodule links, and higher nestedness for more overlaps of partnership, while they tend to split beyond $\Omega_{m,T}$, which inversely increases modularity and decreases nestedness. Thus, the measures Q and $NODF$ show a concave and convex dependence on the intensity of mutualism Ω_m, respectively (Fig. 2c and 2d in the main text). On the other hand, enhancing competition drives modules to merge (see Supplementary Fig. 10b), which decreases modularity Q and increases nestedness $NODF$ monotonically (as shown by coloured curves in Fig. 2c and 2d in the main text). Competition may build up a lumped distribution of species abundances due to competitive repulsion [8]. Increased module sizes, rendering a larger separation between module hubs (generalists with high abundances), would thus be favoured to counteract intensified competition. Hence, the above shows an optimal linking strategy by adjusting all modules as a whole. Due to such systematic change of multiple factors (module sizes, intermodule links and overlaps of partnership), nestedness and modularity remain negatively correlated if the network is at the optimal steady state.

Such structural adjustment is also reflected in the niche complementarity among partner species [18, 19]. We

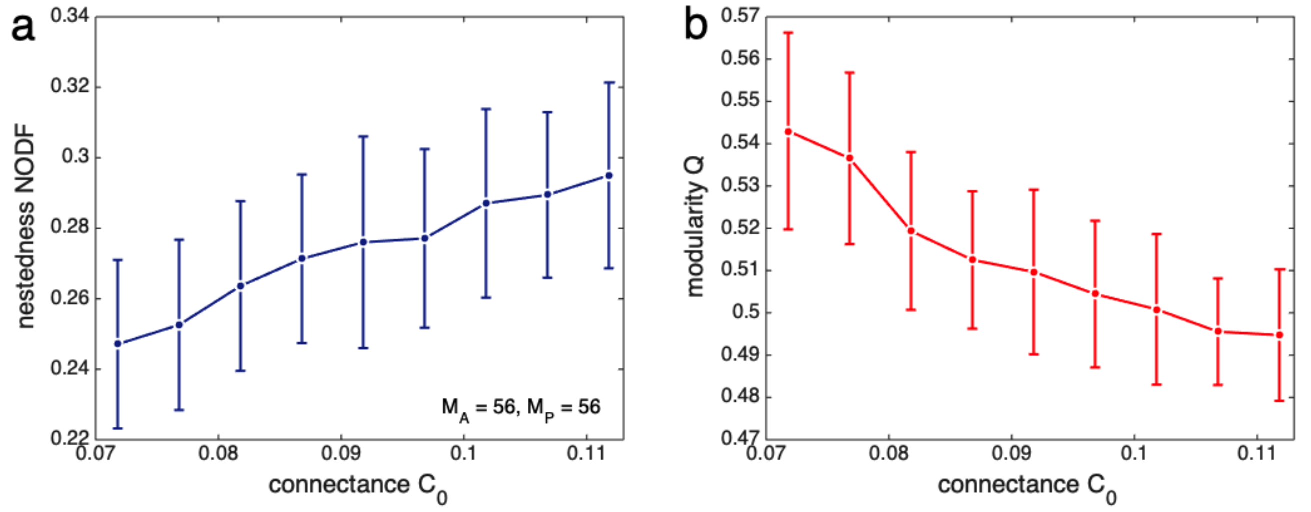

Supplementary Figure 9. Dependence of **(a)** nestedness $NODF$ and **(b)** modularity Q on the connectance C_0 with a fixed network size M. C_0 changes within the vicinity of $\langle C_0 \rangle = 4/M^{0.8} = 0.0918$ with M fixed at 112. The parameters are chosen as follows: $\Omega_m = 0.05$, $\Omega_c = 0.05$ and $\sigma = 0.2$. Data are obtained from 25 simulation runs and presented as mean values $\pm$ SD.

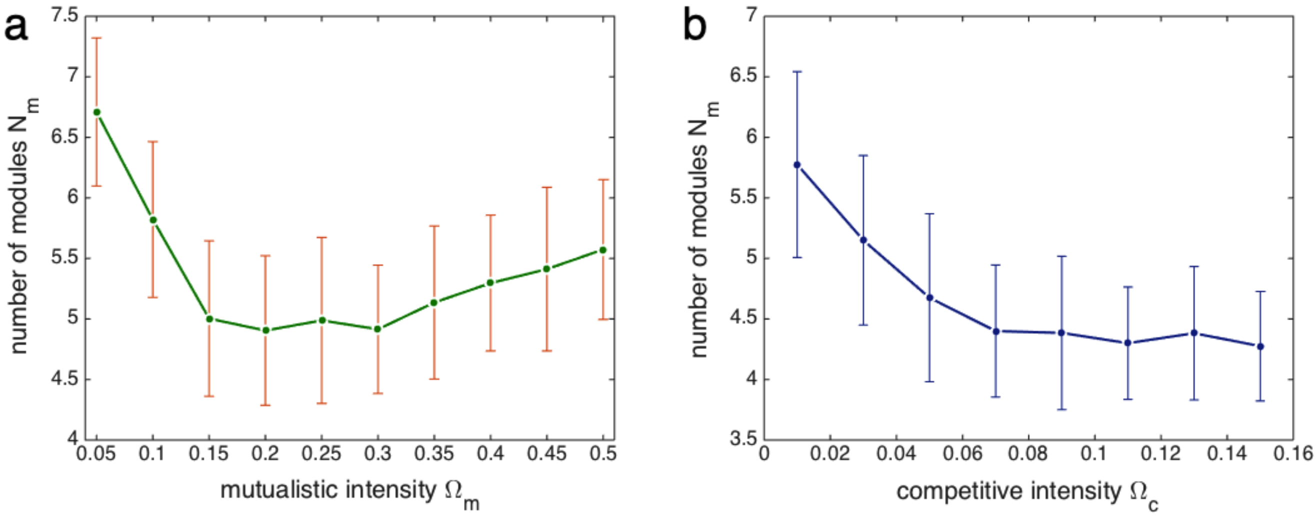

Supplementary Figure 10. Merging and splitting of modules. **a**, Concave dependence of number of modules N_m on mutualistic intensity Ω_m (simulated for $\Omega_c = 0.017$), corresponding to a change from merging to splitting of modules. **b**, Decreasing number of modules N_m with competitive intensity Ω_c (simulated for $\Omega_m = 0.10$) shows a monotonic tendency of merging modules. The niche width is chosen to be $\sigma = 0.1$. Data are obtained from 25 simulation runs and presented as mean values $\pm$ SD.

generate an ensemble of networks by randomly varying the interaction intensities Ω_m and Ω_c (see SI). We define the pairwise niche distance d_{ij} as the separation of niche centers $\bar{s}_i$ and $\bar{s}_j$. The average niche distance $\langle d \rangle$ over all connected species pairs, which reflects the overall niche complementarity, is found to be negatively correlated with the modularity Q and positively correlated with the nestedness $NODF$, as shown in Supplementary Fig. 11. It implies that a more modular structure is packed with more complementary species (lower $\langle d \rangle$), while a more nested structure can tolerate more estranged interspecific partnerships (higher $\langle d \rangle$) [18, 19].

Bounded structure-population relation. The adaptation process is driven by a positive feedback of cumulative advantages in fitness. Locally it realizes the causality between individual species fitness and local structure that a species with a higher abundance would attract species with a broader range of niches. However, no coherent relation exists between the global structure (modularity or nestedness) and the overall population of the community $\Sigma_i n_i$ (equivalently the average abundance per species $\langle n \rangle$). Rather, the structural measures are approximately bounded against the mean abundance $\langle n \rangle$ (Supplementary Fig. 12), suggesting that relatively high nestedness or low mod-

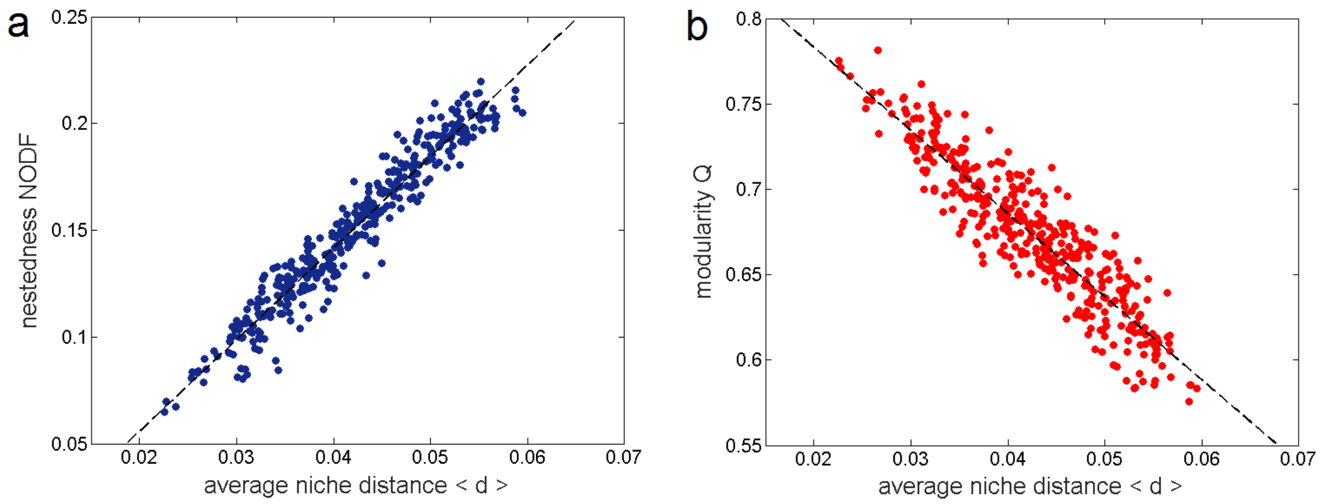

Supplementary Figure 11. Correlation of (**a**) nestedness and (**b**) modularity and average niche distance $\langle d \rangle$ of interacting (linked) species. The data points consists of 300 assembled networks, generated for randomly chosen interaction factors Ω_m and Ω_c, which are fitted with linear relations. The scatter plots consists of 300 assembled networks at feasible equilibria, which are generated for randomly chosen interaction intensities Ω_m and Ω_c in the range $[0.01, 0.50] \times [0.01, 0.10]$, and the niche width is chosen to be $\sigma = 0.1$.

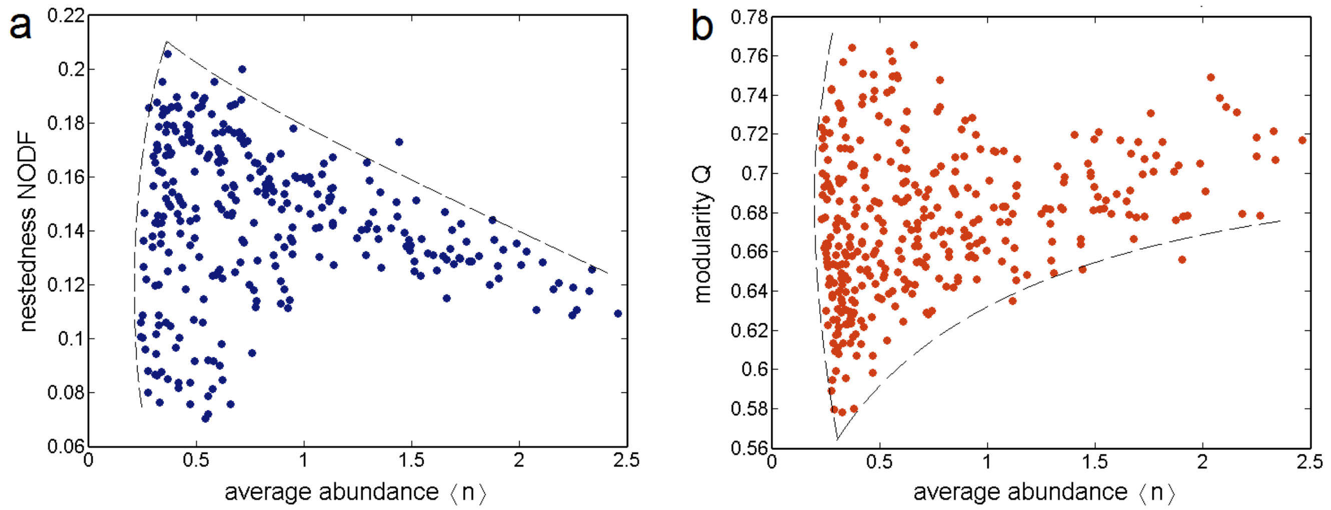

Supplementary Figure 12. Bounded relation of network structure versus average population per species. Structures of relatively high nestedness (**a**) and low modularity (**b**) can be reached only in the region of low mean abundances $\langle n \rangle$. The data points are generated as for Supplementary Fig. 11. The dashed curves are a guide to the eye.

ularity is achievable only when the overall population is low. The relation is demonstrated for an ensemble of 300 generated networks for randomly chosen interaction intensities. This implies that although the mutualistic network structure is formed by maximizing individual species abundances, being more nested or modular does not contribute monotonically to the overall population. The prior claim that greater nestedness facilitates greater population is thus valid only under special conditions [4, 20].

Role of niche width. The niche width σ, which can be interpreted as the intrinsic occupation range of a species on the niche axis, has a strong impact on the structural properties. For simplicity, a uniform value is used to represent the average niche width. Similar to the interaction intensity, the niche width regulates the interspecific interaction pattern through its direct association with the niche proximity. The sizes of modules increase with the niche width and thus allow a more hierarchical structure within each module. Consequently, the nestedness and modularity exhibit a positive and negative correlation with the niche width σ, respectively, as shown in Supplementary Fig. 13a.

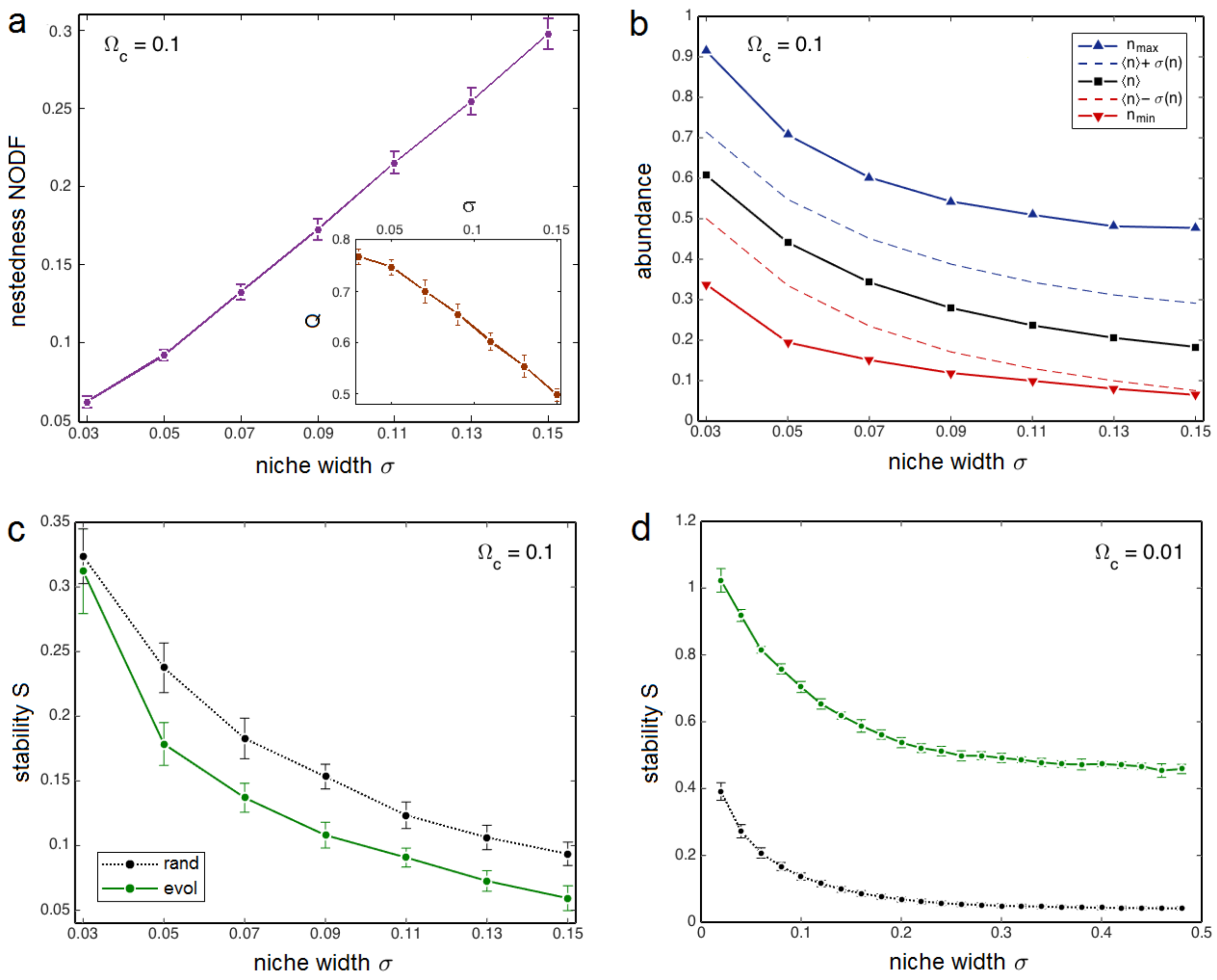

Supplementary Figure 13. Role of niche width. **a**, Structural change. The nestedness and modularity (inset) exhibit a positive and negative correlation with the niche width σ, respectively. For simplicity, all species are assigned the same niche width. **b**, Monotonic change in population. For any competition intensity Ω_c, broadening the niche width always shifts down the entire population distribution, including the average population and the lower and higher bounds of the community. We show here the case for $\Omega_c = 0.1$, but similar tendencies are exhibited for other values of Ω_c. **c, d**, The local stability S consistently decreases with the niche width σ, irrespective of the competition intensity Ω_c. However, the competition intensity still determines the relative stability: the network is consistently less and more stable than the randomized networks (dashed lines) for $\Omega_c = 0.1$ and 0.01, respectively. Data are obtained from 50 simulation runs and presented as mean values $\pm$ SD in panels a, c an d.

We will further address its impact on the dynamical properties in Supplementary Note 3.

SUPPLEMENTARY NOTE 3: DYNAMICAL PROPERTIES AT ECOLOGICAL TIMESCALE

We are now able to examine dynamical properties of mutualistic networks considering both population and structural adaptations. We are most concerned with the network resilience, which reflects to what extent the established macroscopic characteristics are maintained under external influence. Extending the discussions in the main text, we analyze the influences at both ecological and evolutionary timescales in this and next notes.

At a sufficiently short timescale, we assume that the interaction intensities do not vary during a transitory perturbation, so that the network topology remains fixed at the steady state. Such perturbation is associated with the ever-existing small stochastic environmental changes in the species abundances. This allows us to use the real part of the leading eigenvalue of the Jacobian matrix of the network dynamics (Supplementary Eq. 4) as the measure of the local stability, that is, $S = -Re(\lambda)_{max}$. This value is always calculated at the end of the time interval T when the network settles to the equilibrium. Since most rewiring attempts are rejected at the steady state, the stability measure S only fluctuates around a constant value with a small variation (see Supplementary Fig. 5b).

Local stability. At equilibrium, the Jacobian Φ adopts a relatively simpler form, since the right hand side of Supplementary Eq. 4 equals zero.

$$\Phi = \begin{bmatrix} \Phi^{AA} & \Phi^{AP} \\ \Phi^{PA} & \Phi^{PP} \end{bmatrix} = \begin{bmatrix} \partial \dot{n_A}/\partial n_A & \partial \dot{n_A}/\partial n_P \\ \partial \dot{n_P}/\partial n_A & \partial \dot{n_P}/\partial n_P \end{bmatrix} \tag{7}$$

where the components can be written as (symmetric for Φ^{PP} and Φ^{PA})

$$\Phi^{AA} = -\text{diag}(n_A) \cdot \beta^A \tag{8}$$

$$\Phi^{AP} = -\text{diag}(n_A) \cdot \xi^{AP} \tag{9}$$

with

$$\xi^{AP}_{ij} = \frac{\left[\gamma^{AP} + h \cdot \text{diag}(\rho^A - \beta^A n_A)\theta^{AP}\right]_{ij}}{(1 + h \cdot [\theta^{AP} n_P]_i)} \tag{10}$$

The symbol diag(v) denotes an $L \times L$ diagonal matrix whose diagonal entries are the elements of the vector $v \in \mathbb{R}^L$. We have nummerically calculated the eigenvalues for all figures, according to Supplementary Eq. 7 - 10. However, following the perturbation expansion [4], in the limit when both γ_{ij} and β_{ij} $(i \neq j)$ are far smaller than the diagonal entries β_{ii} (self-limiting coefficient), the eigenvalue of the Jacobian is predominantly determined by the abundances at equalibrium, that is, $\lambda_i \approx -n_i \cdot \beta_{ii}$. Hence, the stability measure is approximately only related to the minimum abundance of the poorest species in the community $S \approx -n_{min} \cdot \beta_{ii}$ (we normalize the diagonal entries $\beta_{ii} = 1$). This simple relation links the stability of the community and the lower bound of the population (see inset of Supplementary Fig. 17a).

Scaling and aspect-ratio effects on stability. We have shown in Supplementary Note 2 the structural dependence on mutualistic and competitive intensities, which are robust for different system sizes M and aspect ratios R_M (Supplementary Fig. 7 and 8). Following the same routine, we examine the impact of M and R_M on the local stability S. As shown in Supplementary Fig. 14, the network is destabilized by increasing either the system size M or R_M. The destabilization in both cases is due to the fact that the competitive repulsion escalates when the density of species within one or both guilds increases. However, enhancing mutualism by Ω_m can still either stabilize or destabilize the network at the stead state, determined crucially by the competition intensity Ω_c. This is demonstrated by the existence of both rising and falling tendencies of S in Supplementary Fig. 15 (different M) and 16 (different R_M). This scenario of competition-controlled stability supports the general conclusion on the bidirectional role of mutualism as stated in the main text.

Bounded stability-structure relation. The intriguing question is how the pervasively observed nested or modular patterns influence the network stability, which has aroused substantial interest. In our adaptive niche model, both network structure and the stability change with the interaction intensities (Fig. 2c, 2d and Fig. 3a-3c). However, the stability S shows only a bounded negative and positive tendency with nestedness $NODF$ and modularity Q, respectively (see Supplementary Fig. 17). This is demonstrated for generated networks corresponding to randomly chosen interaction intensities Ω_m and Ω_c. The network structure influences the entire population distribution, but does not directly regulate the lower bound of abundance n_{min}. A simple correlation of the stability and any structural measure is thus absent.

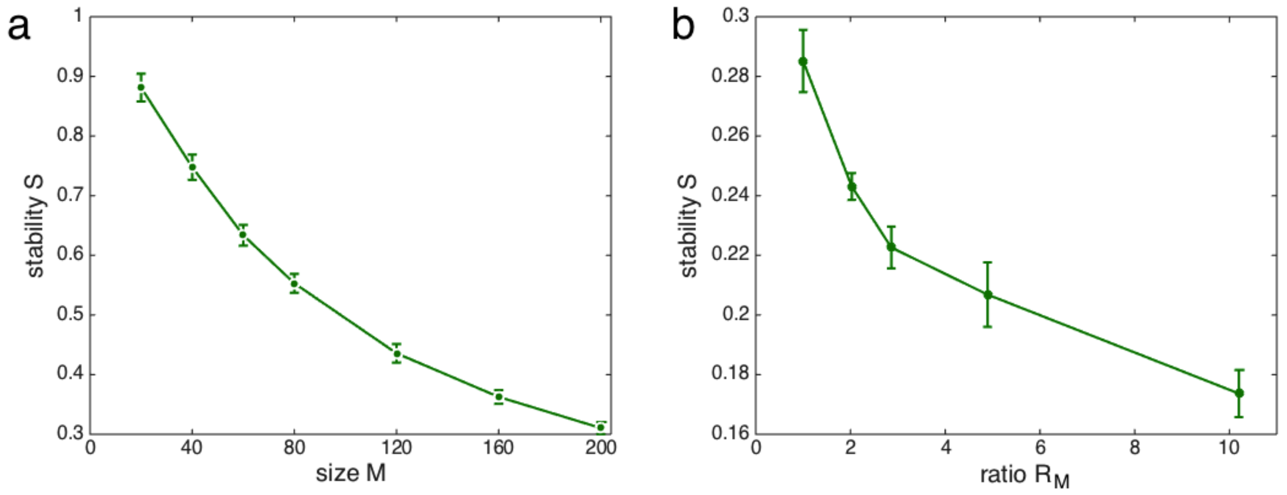

Supplementary Figure 14. Stability versus (**a**) network size M and (**b**) aspect ratio R_M. **a**, M ranges in [20, 200] with a fixed $R_M = 3$. **b**, R_M ranges in [1.0, 10.2] with a fixed $M = 112$. For all data points, the parameters are chosen as follows: $\Omega_m = 0.05$, $\Omega_c = 0.05$ and $\sigma = 0.2$. The connectance is set to be a function of the network size $C_0 = 4/M^{0.8}$. Data are obtained from 25 simulation runs and presented as mean values ± SD.

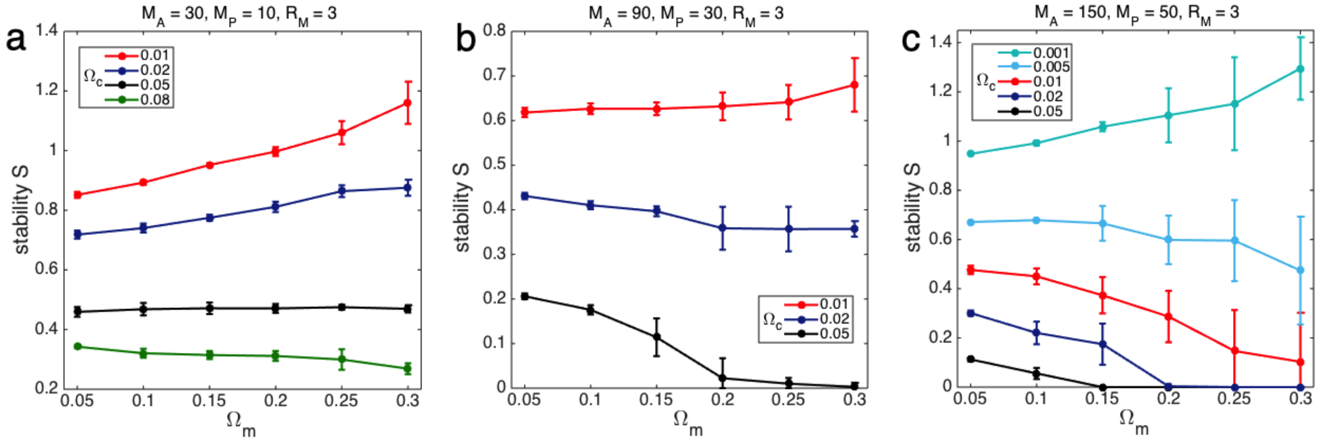

Supplementary Figure 15. Stability versus interaction intensities for different network sizes $M = M_A + M_P$ (**a,** $M = 40$, **b,** $M = 120$, **c,** $M = 200$) with a fixed aspect ratio $R_M = M_A/M_P = 3$. The stability S has either a positive or negative dependence on the mutualistic intensity Ω_m, which is determined by the competition intensity Ω_c. The niche width is chosen to be $\sigma = 0.2$. Data are obtained from 25 simulation runs and presented as mean values ± SD.

Roles of competition and mutualism on stability. We have identified the crucial role of competitive interaction on stability in the main text. The competition intensity determines whether enhancing mutualism would stabilize or destabilize the network. The contrasting tendencies of stability are revealed in the population distribution through the relation between the minimum abundance n_{min} and stability S (Supplementary Fig. 18). In concrete, for Ω_c above $\Omega_{c,T}^{II}$, the average abundance increases slightly but the distribution is broadened, with both highest and lowest limits extending in the opposite directions; for Ω_c below the transition point $\Omega_{c,T}^{II}$, the entire distribution shifts towards greater abundance with increasing Ω_m.

The role of mutualism is thus highly differentiated on the community: enhancing mutualism is always beneficial for the "rich" species, but can be less beneficial or even detrimental for the "poor" species. More than that, enhancing mutualism is prone to dividing the hierarchies. Species become concentrated on a multi-modal population distribution when the mutualism is intensified (lower rows of Supplementary Fig. 18). The lowest hierarchy, which determines the stability of the network, is most subject to the competition intensity.

We further examine the impact of niche width on network stability. Increasing the niche width σ always destabilizes the network, independent of the competition intensity Ω_c (see Supplementary Fig. 13c and 13d). This is again consistent with the lower bound n_{min} of the species abundances. In fact, the entire population distribution shifts

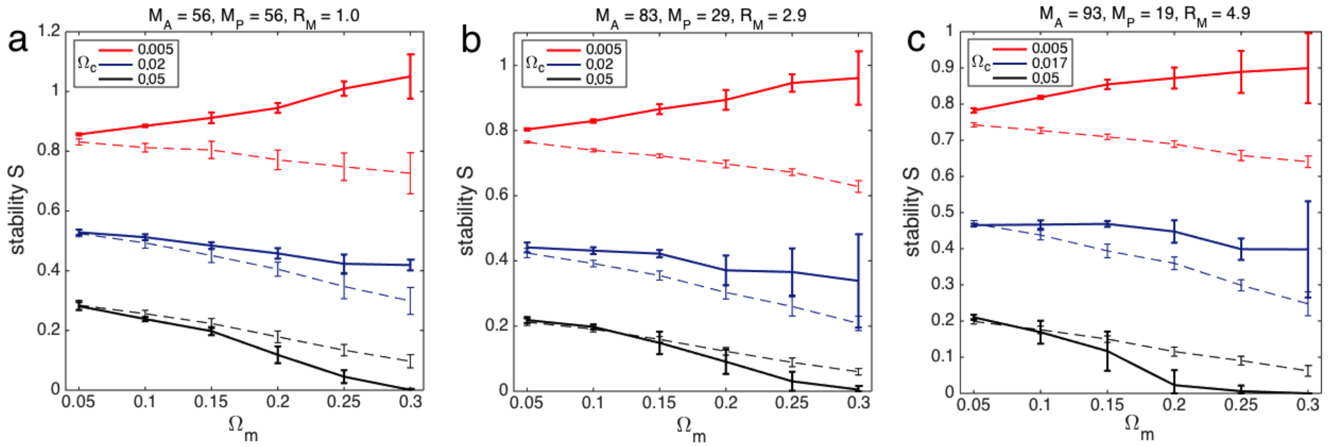

Supplementary Figure 16. Stability versus interaction intensities for different aspect ratios $R_M = M_A/M_P$ (**a,** $R_M = 1.0$, **b,** $R_M = 2.9$, **c,** $R_M = 4.9$) and a fixed network size $M = M_A + M_P = 112$. The stability S has either a positive or negative dependence on the mutualistic intensity Ω_m, which is determined by the competition intensity Ω_c. The stability measures for the null model are shown by the dashed curves. The niche width is chosen to be $\sigma = 0.2$. Data are obtained from 25 simulation runs and presented as mean values ± SD.

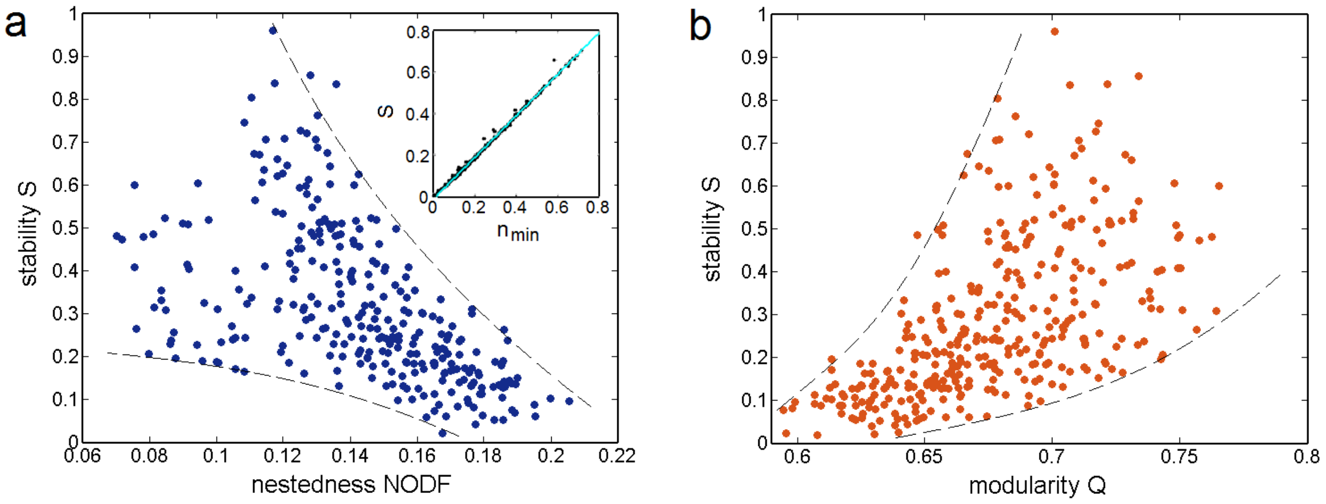

Supplementary Figure 17. Bounded relation between stability and structure. The network stability S shows an approximately bounded negative and positive dependence on the nestedness $NODF$ (**a**) and modularity Q (**b**), respectively. Inset: The stability S is correlated with the lower bound of the species abundance in the community, through the simple relation $S \approx n_{min}$ when the interspecific interaction is far less intensive compared with the intraspecific competition. The networks analyzed here are randomly generated as for Supplementary Fig. 12.

persistently down with the niche width σ for any Ω_c (Supplementary Fig. 13b). It suggest that all species are less benefited from mutualism when the interaction range increases but the niche proximity per pair of species decreases.

History-dependence for small-sized networks. As we have shown in the main text, the structural adaptation of mutualistic network is asymmetric, exhibiting hysteretic trajectories in the ascending and descending paths with the mutualistic intensity Ω_m in response to environmental change. The history-dependence is intrinsic to the system due to the fact that the preferential attachment process prefers in the direction of merging modules rather than splitting them. However, this effect is less prominent in a smaller-sized network, as we show in Supplementary Fig. 19 that the trajectories in opposite directions are less distinguishable when M is reduced to 112, compared to those for $M = 200$ shown in Fig. 4 of the main text. This demonstrates a strong finite size effect on the structural adaptation.

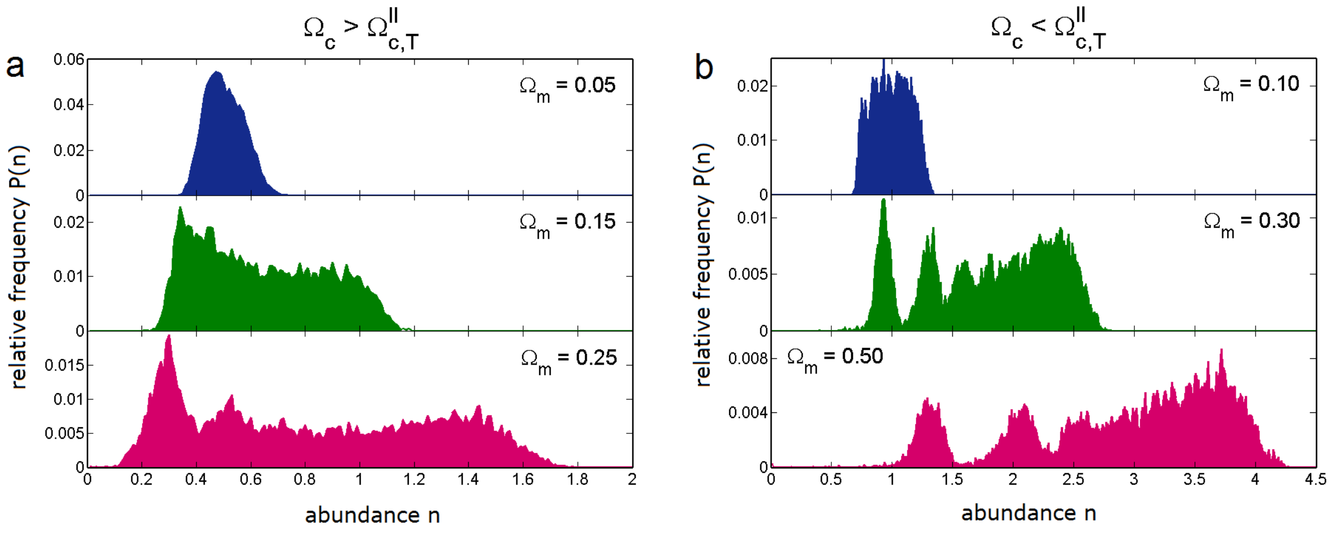

Supplementary Figure 18. Contrasting modes of changes in population distribution. **a**, For $\Omega_c = 0.035 > \Omega_{c,T}^{II}$, the distribution becomes substantially broader with the intensity of mutualism Ω_m, with both highest and lowest limits extending in the opposite directions. **b**, For competitive intensity $\Omega_c = 0.01$ below the transition point $\Omega_{c,T}^{II}$ (≈ 0.015), the entire distribution shifts up with Ω_m. The population distribution are multi-modal at high Ω_m, indicating that enhancing mutualism is prone to dividing the species into hierarchies. Each bin width of the histogram is 0.01.

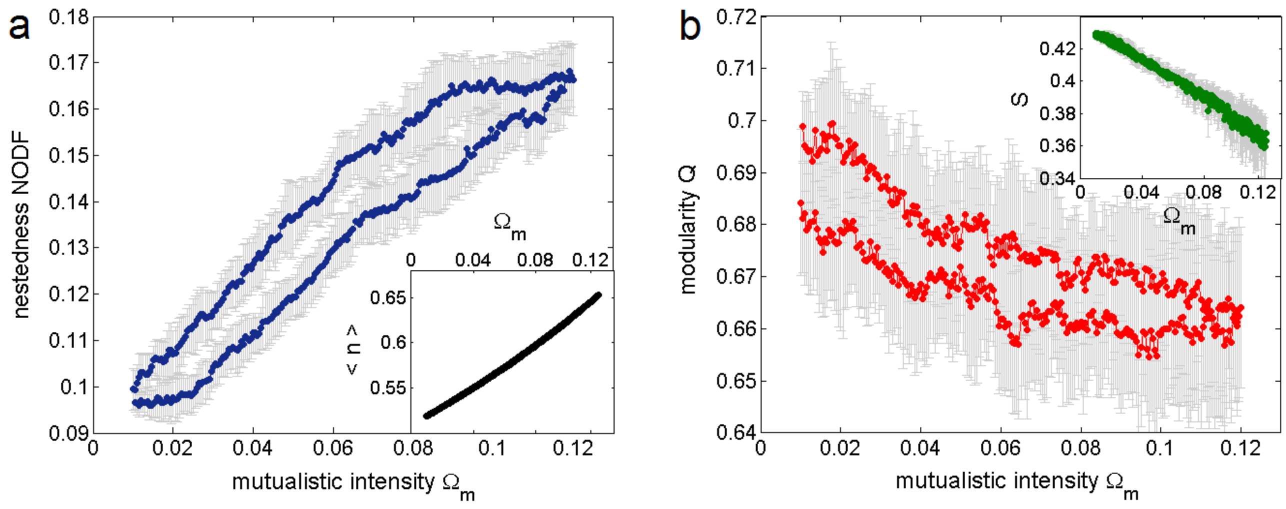

Supplementary Figure 19. Structural adaptation (**a**, NODF, **b**, Q) with mutualistic intensity Ω_m for networks of relatively small sizes. The numbers of species are chosen as $M_A = M_P = 56$. The asymmetry in trajectories is exhibited less prominently when the system size is reduced, compared to results shown in Fig. 4 in the main text. A very slow change rate ($\Delta\Omega_m = 10^{-6}$ per time interval) is used by the reciprocation of Ω_m so that the system is guaranteed to be always at quasi-steady-states. Ω_c is fixed at 0.05. The insets show the trajectories of the average abundance per species $\langle n \rangle$ and of the stability S. Data are obtained from 50 simulation runs and presented as mean values $\pm$ SD.

SUPPLEMENTARY NOTE 4: INVASION TO MUTUALISTIC NETWORKS

As discussed in the main text, the adaptive niche-based dynamics plays a critical role in preserving the mutualistic network structure under invasions. This cannot be captured by static network models, in which the network topology does not adapt to the addition of mutant species. Here, we provide more details concerning the invasion dynamics and its impact on the network assembly in this adaptive framework. From stochastic simulations, we show that in the presence of invasions and extinctions, the mutualistic network exhibits persistently a nontrivial architecture with reasonable nestedness and modularity. It suggests surviving mutants find the "right" entry points to the network and thus preserve its particular pattern.

Adaptive-evolutionary dynamics. Unlike the conventional theory of "adaptive dynamics", in which a single evolutionary timescale is considered, we adopt a two-timescale dynamics: adaptive rewiring at a small ecological timescale ($\Delta T_r = T$) and invasions of mutants at a large evolutionary timescale ($\Delta T_m = R_T * T$). The ratio R_T of timescales does not affect the results significantly if it is set sufficiently large (here we set $R_T = 100$). At each time interval ΔT_m, a resident species (already in the network) is randomly chosen with probability that is proportional to the abundnance n_i of the resident. (We also checked the results for choosing the resident species from a uniform probability distribution and the results are qualitatively the same.) A mutant species is created, which comprises of 1% of the resident's total abundance (thus the resident's abundance drops to $0.99n_i$) and inherits all its mutualistic links to the partners. Yet, the mutant deviates from the resident by a displacement on the niche axis [21], which is drawn from a Gaussian distribution, i.e. $\overline{s_{i'}} \sim N(\overline{s_i}, \sigma_m)$ (here σ_m is set to be 0.01). Since the mutant originates from the resident's population, within-guild competition exists between the mutant and the resident with the intensity determined by their niche overlap $\beta_{ii'} = H_{ii'}$. To keep the connectance comparable to that observed in empirical networks $C_0 = 4/M^{0.8}$ (M being the total number of species), we introduce a probability P_d for deletion of extra links throughout the network after the mutation. Any existing link is deleted with $P_d = \max(0, 1 - C_0 \cdot M_A \cdot M_P / N_L)$, where N_L is the total number of links right after the mutation. In this way, we directly simulate the outcome of the invasion from a tiny abundance of the mutants. Any species is removed from the network if its abundance drops below a small threshold $n_c = 10^{-4}$. All possible fates of the mutants and residents can then be observed in the simulation: a mutant can survive or go extinct, and the resident species may persist or go extinct.

An effective way to accelerate the selection of resident species is based on the first-reaction method [22]. At each time interval ΔT_m, one draws M random numbers $r_1, \ldots, r_M$ from a uniform distribution in $[0, 1]$ and compute $\tau_i = \frac{1}{n_i} \ln\left(\frac{1}{r_i}\right)$ for each species. The smallest τ_{i^*} of all $\{\tau_i\}$ is then selected where i^* corresponds to the species at which an invasion is attempted.

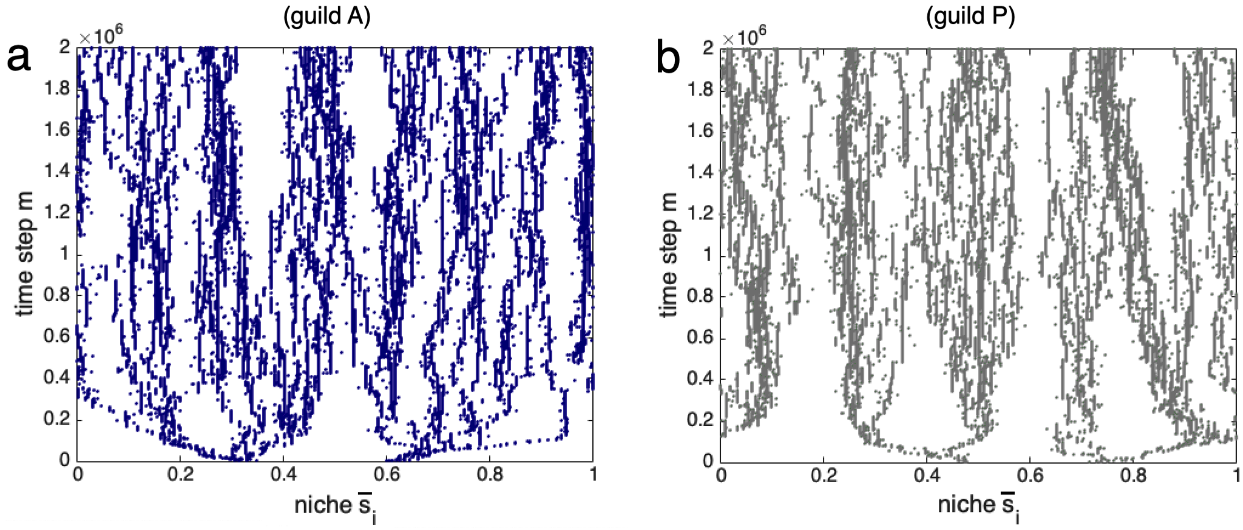

Supplementary Figure 20. Trait evolution by invasion. A pair of initial species in each guild (**a**, A and **b**, P) are placed at randomly selected niche positions. Invasion of mutants is assumed more likely to occur at the resident species with a higher abundance. The trait evolution shows that a mutant can replace the resident, be driven extinct or coexist with the resident, depending on the position of entering the network. The points shown here are sampled every 1000 time steps.

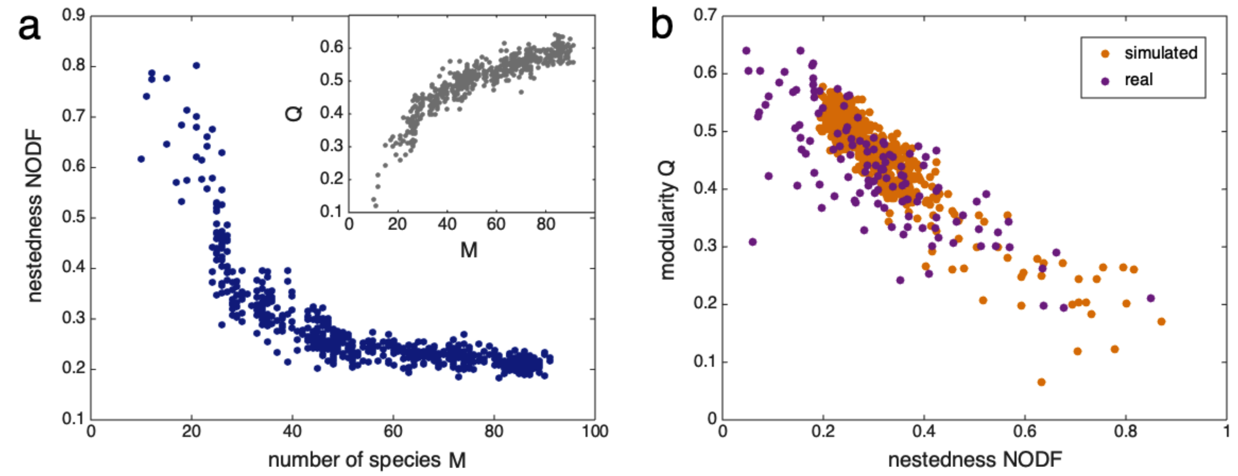

Supplementary Figure 21. Structural change of an example network during repeated invasions. **a**, Nestedness and modularity (inset) show a decreasing and increasing tendency with the number of species M, respectively. **b**, Dyadic structural measures during invasions (shown for one simulated realization under $\Omega_c = 0.5$), compared with the data points of 144 empirical networks from the Web of Life dataset.

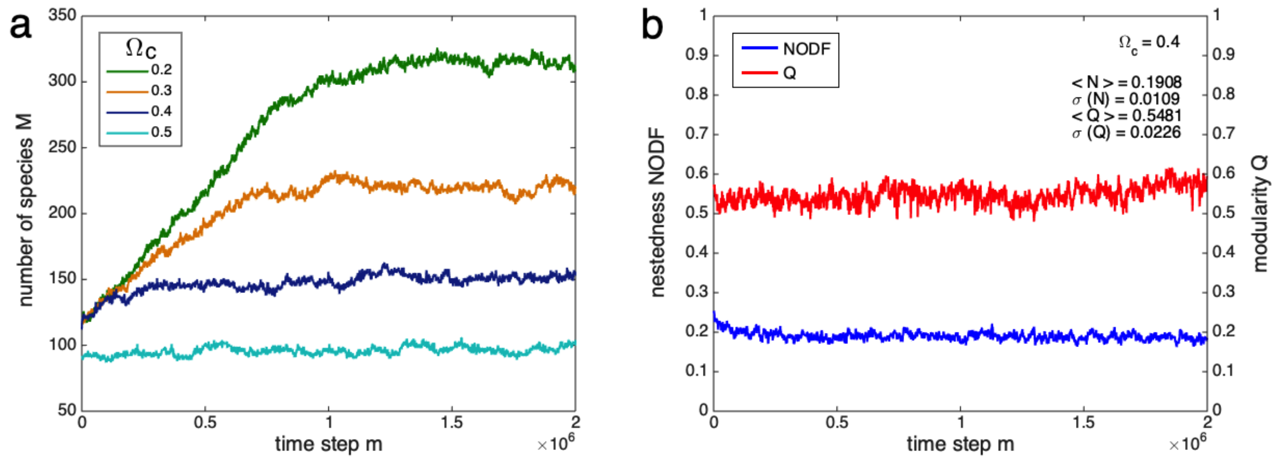

Supplementary Figure 22. Invasion to an existing network. The initial network is consisted of 56 species in each guild, self-organized according to the rules in Supplementary Note 1. **a**, Total number of species under different competition intensities. The starting number for $\Omega_c = 0.5$ is lower than 112 due to limited network capacity. **b**, Nestedness and modularity measures versus time, simulated for $\Omega_c = 0.4$.

We use two initial conditions for invasion tests. The test of the first type starts from a minimal core of resident species, $M = 4$. This core consists of two species in each guild with all cross-guild connections present, thus there is both within-guild competition and cross-guild mutualism at the beginning. The niche positions of the starting species are randomly selected on the niche axis (but these initial niche positions turn out not to affect the long-term statistical outcome). Our numerical simulations show that given sufficient time, mutant species will explore the entire niche space by showing two phylogenetic trees in each guild with each tree rooted at the niche of the initial species, as displayed for example in Supplementary Fig. 20.

As shown in Fig. 5a in the main text, the maximal capacity of the network, reduces with the competition intensity Ω_c. It is notable that when saturation is reached, invasion and extinction are still possible but balanced out. Nestedness decreases and modularity increases with the rise of species number (see Supplementary Fig. 21a). However, throughout the evolution both structural measures show a distribution in the $(NODF, Q)$ plane that overlaps with the band of data points of the empirical networks from the Web of Life dataset (see Supplementary Fig. 21b). It suggests that the simulated network has evolved along a reasonable path due to the adaptive niche-based dynamics, which has preserved the structure during invasions.

We further test invasions to a relatively large network. We start from an existing nested and modular structure (here

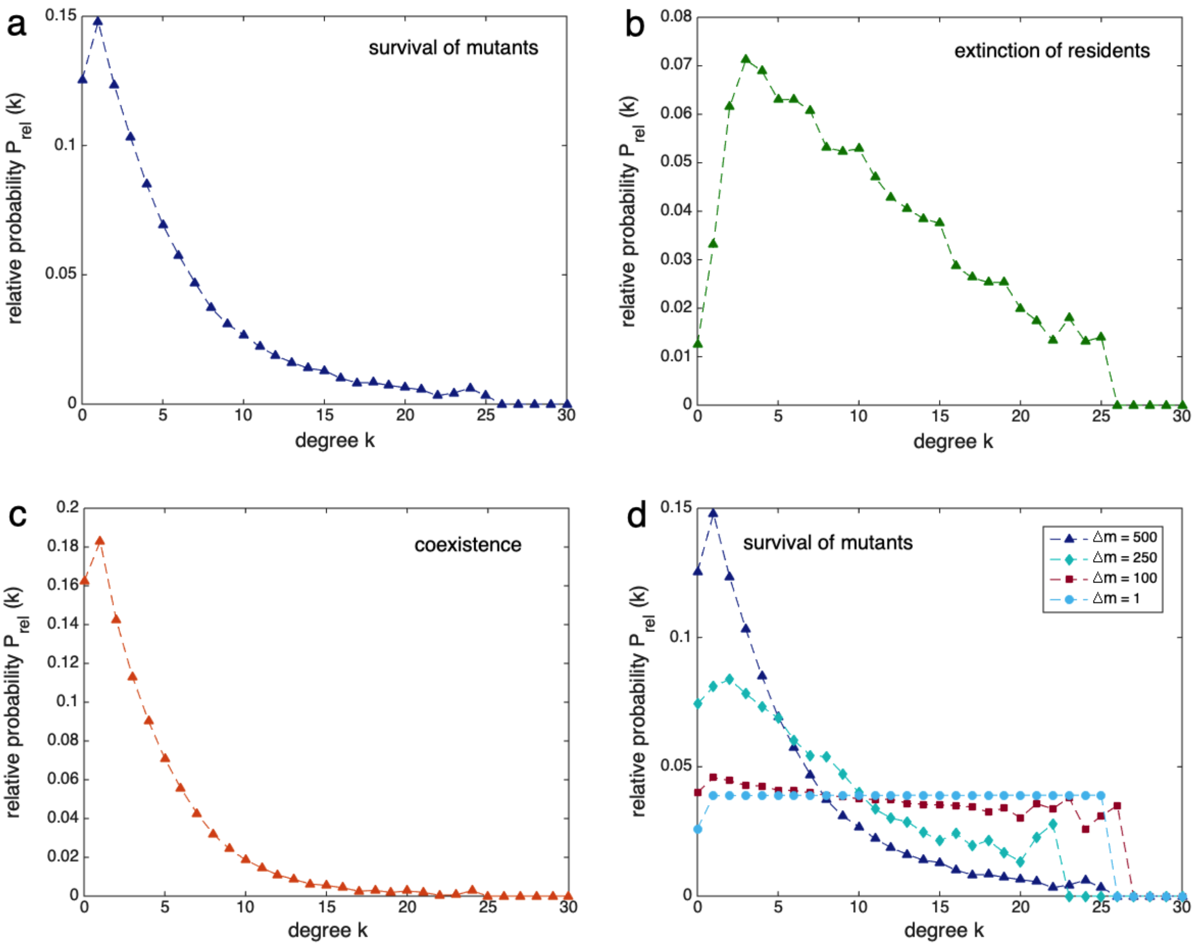

Supplementary Figure 23. Relative probabilities for invading mutants and invaded residents versus the degree k in the adaptive-evolutionary dynamics. **a**, Probability that a mutant survives. **b**, Probability that an invaded resident goes exist (peak at $k = 3$). **c**, Probability that a resident and its mutant coexist. These three panels are obtained by examining the outcome of mutants and residents $\Delta m = 500$ time steps after the invasion. **d**, The relative probabilities of the survival of the mutant at different time points ($\Delta m = 1, 100, 250, 500$) after the invasion.

we set $M_A = M_P = 56$) that is self-organized according to the rules given in Supplementary Note 1 and introduce invasions thereafter. Again, we observe saturation of the network under competition, as shown in Supplementary Fig. 22a. But the nestedness and modularity change merely slightly due to the weak dependence of structural measures on the species number when the latter is high (see Supplementary Fig. 22b). The stable structure provides us an ideal setting for studying the relation between the survival and extinction probabilities and the network structure.

This preservation of the network structure is achieved because the adaptive dynamics results in different resilience of the actors. We calculate the relative (conditional) probability [23] $P_{rel}(k)$ that a mutant has survived for Δm steps given that the selected resident has a degree k (see Supplementary Fig. 23a, where $\Delta m = 500$). We calculate first the absolute distribution $P(k)$ of surviving Δm time steps after invasion to residents with degree k, and then normalize it by the degree distribution $Q(k)$ of the selected residents. The result is averaged over 100 simulation runs and 10 different initial networks. Similarly, we calculate the relative probability that a resident has gone extinct (Supplementary Fig. 23b) and that both resident and mutant coexist (Supplementary Fig. 23c) Δm time steps after the invasion. All three relative probabilities tend to decrease with the degree of resident (except some low degrees), following exponential distributions (see also Fig. 5c in the main text). The skewed distributions show that the cores of the modules, comprised of generalists (high-degree nodes), are seldom affected by invasions while the peripheral specialists (low-degree nodes) are substituted more frequently.

Note that although the mutant is always initialized with a tiny abundance at the beginning (so does not die out instantaneously), its fate can change over time as the adaptive rewiring precedes to optimize the network structure. This is in contrast with the conventional Adaptive Dynamics in which the fate of the mutant can be judged right

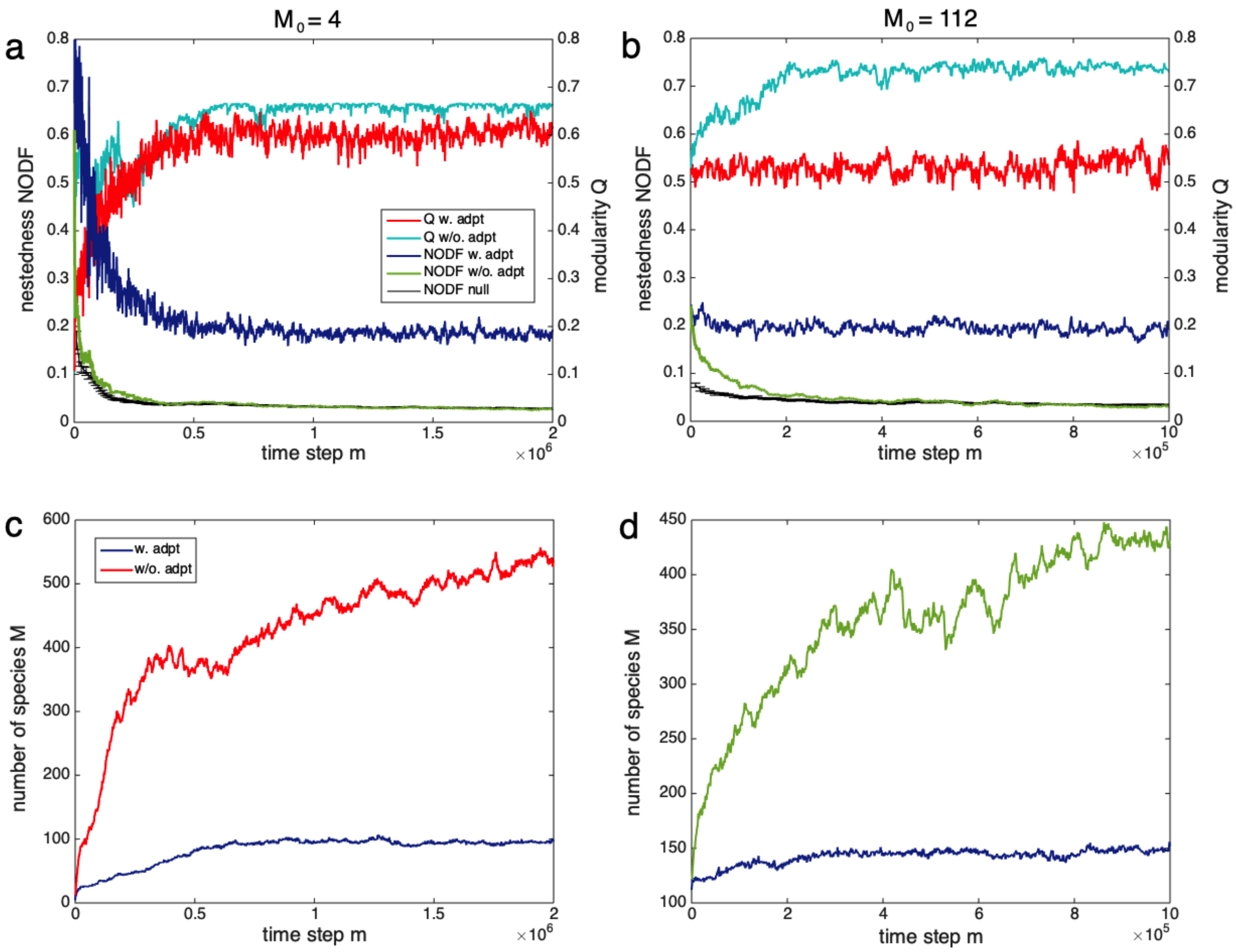

Supplementary Figure 24. Adaptation preserves nestedness under invasion. The time evolution of structural measures are shown for the invasion process with and without adaptation, starting from an initial network of size **(a)** $M_0 = 4$ ($M_{0,A} = M_{0,P} = 2$) and **(b)** $M_0 = 112$ ($M_{0,A} = M_{0,P} = 56$). Without adaptation, the nestedness $NODF$ decreases rapidly to a value comparable to that of the null model, while the modularity Q remains (increases) due to competitive repulsion. We use random networks that preserve the same network size M and connection c_M as the null model for comparing the nestedness at the corresponding time step (the error bars represent one standard deviation). **(c)** and **(d)**, bounded versus unbounded network size with and without adaptation under invasion. The network reaches a plateau at around the size $M = 100$ and $M = 150$ with adaptation, starting from $M_0 = 4$ and $M_0 = 112$, respectively.

after the invasion (by the initial growth rate) since the environment does not change [24–26]. Therefore, one needs to judge the survival or extinction of the mutant after different lengths of time lapse Δm after a mutation. For example, the relative probability for a mutant to survive Δm time steps has a uniform distribution against the degree of the invaded mutant immediately after the invasion ($\Delta m = 1$), but becomes more skewed when Δm increases, as shown in Supplementary Fig. 23d. It implies that mutants which have invaded generalist resident species are more likely to go extinct as the adaptation of the network continues.

It is important to note that for the evolutionary process without adaptation (discussed in the next section), the relative probability for the survival of a mutant is instead unbiased, regardless of the time lapse Δm. As shown in Supplementary Fig. 25 the relative probability for a mutant to survive Δm time steps is independent of the degree of the resident node to be invaded (shown for $\Delta m = 500$). So, in contrast to the adaptive-evolutionary process, mutants invading residents of any degree can equally survive.

Evolution without adaptation. To establish that the adaptive process is crucial for preserving the mutualistic network patterns, we make a comparison by studying the evolutionary invasion process with no adaptive rewiring involved. Figure 24a and 24b show that with the adaptive process absent, the nestedness $NODF$ quickly decays to a low level comparable to that of a corresponding random network with the same network size and connectance, yet

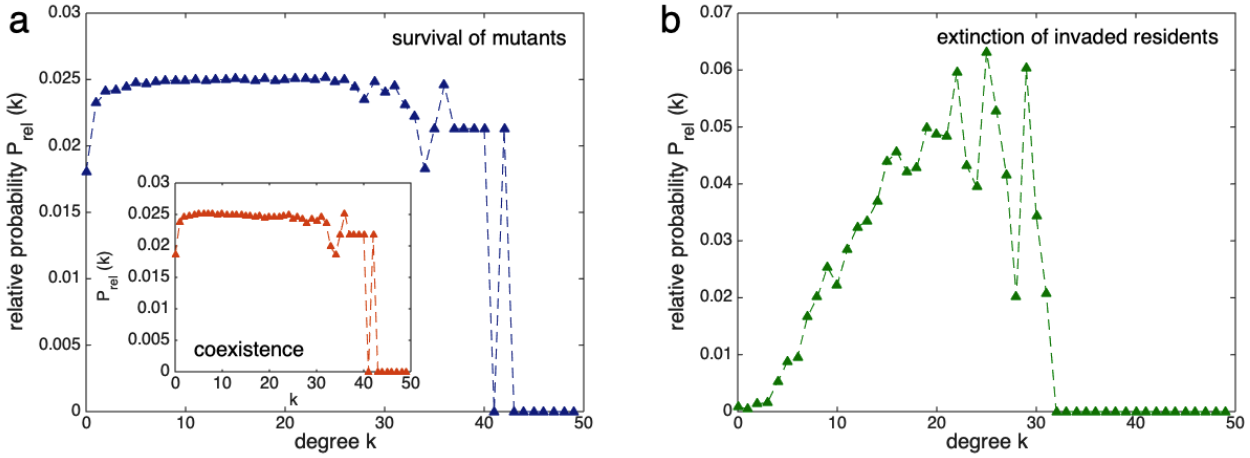

Supplementary Figure 25. Relative invasion probabilities versus degree k of the resident species under invasions with no adaptation involved. **a**, Probability that a mutant has survived $\Delta m = 500$ steps after the invasion. Inset: Probability that a resident and its mutant coexist. Both show no dependence on the degree (before the cut-off). **b**, Probability that a resident has gone exist within Δm time steps after the invasion of a mutant, which increases generally with the degree of the resident.

the modular structure remains due to the underlying mechanism of limiting similarity from competitive repulsion [8]. Figure 24a shows this for the invasion of a small network ($M_0 = 4$), and Supplementary Fig. 24b for the invasion of a large network ($M_0 = 112$). Without the adaptive dynamics the network size increases unboundedly, as demonstrated by the comparison to the process with adaptation shown in Supplementary Fig. 24c and 24d (with $M_0 = 4$ for (c) and $M_0 = 112$ for (d)). Note that excessively large sizes are seldom observed in empirical mutualistic networks [13].

As shown in the previous section, the adaptive-evolutionary dynamics create a degree-based bias in the survival probability, shown in Supplementary Fig. 23. But, without the adaptive dynamics, the relative probabilities of survival and of coexistence are unbiased and relatively uniform for all degrees of the resident species, while the relative probability of the resident being driven extinct generally increases with the resident's degree (see Supplementary Fig. 25). This leads to the rapid decay in nestedness shown in Supplementary Fig. 24a and 24b.

Limiting similarity. It is noteworthy that the phenomenon of limiting similarity is ubiquitously observable in systems involving niche-based competition [8]. In the adaptive-evolutionary process, we have also observed that the traits of species, represented by their niche positions, tend to converge into multiple humps (Supplementary Fig. 26). This is consistent with niche shift due to the competitive repulsion discussed by Scheffer and van Nes [8]. Yet, niche shift contributes only to establishing modules but, on it's own, it cannot lead to the nested pattern. The role of adaptation in evolution is thus indispensable for establishing the nontrivial architecture.

An important remark is that despite the difference in the nature of the interactions, the emergence of modular structure is intrinsically similar to that in the evolutionary mechanism describing predator-prey relations, where stratification of trophic levels also has a crucial dependence on the niche-based interactions [27]. Such common behaviour can trace back to the optimization principle of niche dynamics of coexisting species studied by MacArthur and Levin [2, 8].

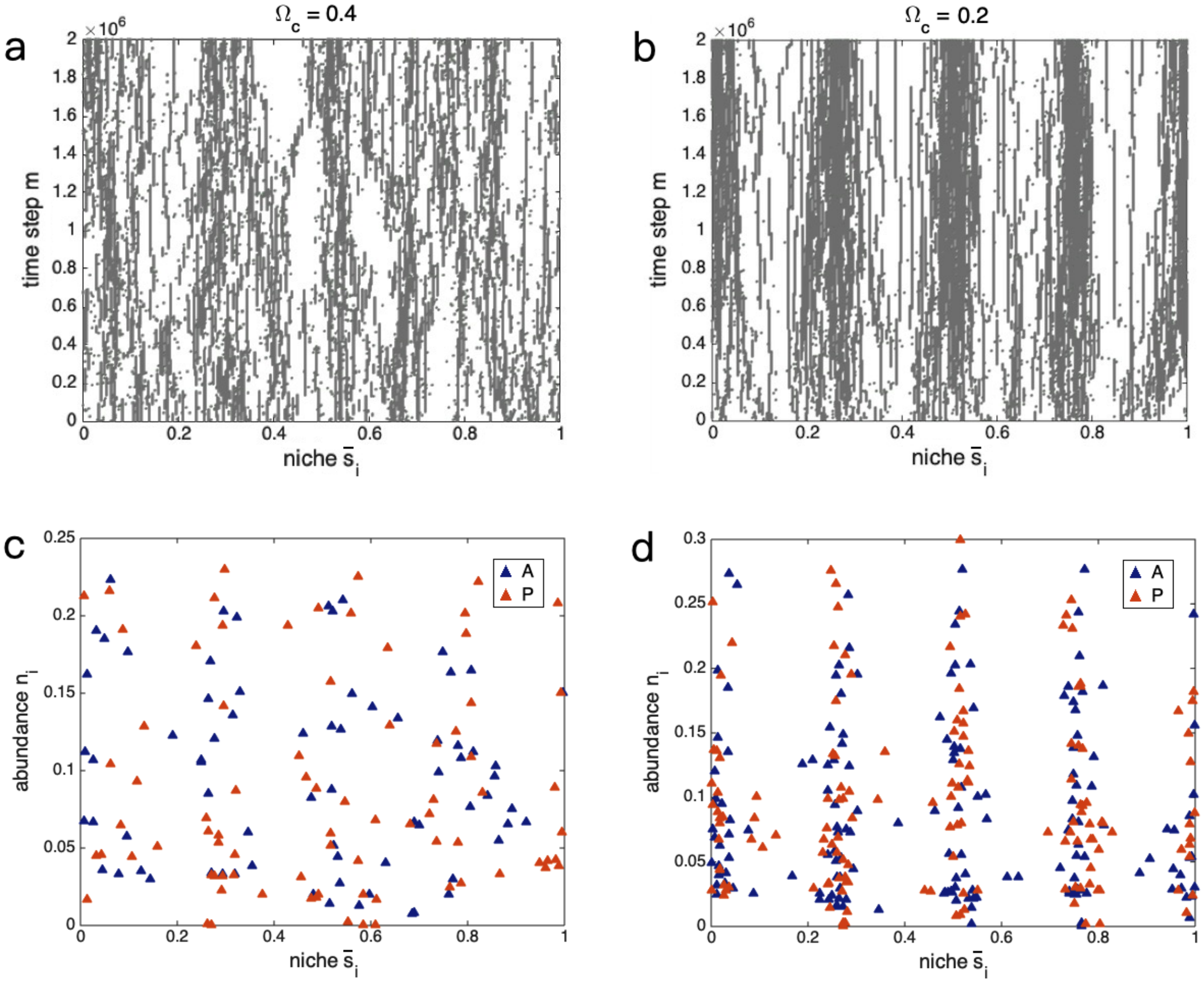

Supplementary Figure 26. Limiting similarity in the adaptive-evolutionary process. **a, b**, The phenomenon of lumped niches is more prominent under lower competition intensity Ω_c, for which more species are allowed in the network. **c, d**, Abundance of species n_i versus their central niche positions $\overline{s_i}$. The results are shown for the case of invasions to an existing large network of 112 species, but similar patterns are observable for a growing network from a small core. The effect of limiting similarity is more prominent in panel (d) with lower Ω_c, where more species can coexist in the network. Panels (a) and (c) are simulated for $\Omega_c = 0.4$; (b) and (d) are simulated for $\Omega_c = 0.2$.

SUPPLEMENTARY NOTE 5: SUBOPTIMAL STRUCTURE

The optimization process may be accompanied by imperfectness due to stochastic fluctuation, known as the noise effect, which can lead to a suboptimal network structure. Instead of finding specific reasons, one can generally model the imperfectness by introducing a noise in the process, as a convention used in statistical physics (such as in Ising model and simulated annealing process [28, 29]). In our model, we can also suppose that the rewiring probability $P(\theta_{ik} \to \theta_{ik'})$ is affected by a noise level, which is parameterized by a virtual temperature $\mathcal{T}$

$$P(\theta_{ik} \to \theta_{ik'}) = \begin{cases} 1, \text{if } n_i(t+1;\theta_{ik'}) > n_i(t;\theta_{ik}) \\ \exp\left[-\left(n_i(t;\theta_{ik}) - n_i(t+1;\theta_{ik'})\right)/k_T\mathcal{T}\right], \text{otherwise} \end{cases} \tag{11}$$

where k_T is a rescaling coefficient. Note that when $\mathcal{T} \to 0$, it recovers the original model with zero probability for accepting a rewired link that reduces the species abundance n_i. For any finite $\mathcal{T} > 0$, both nestedness and modularity measures decrease with $\mathcal{T}$ (Supplementary Fig. 27). Beyond a critical point $\mathcal{T}_c$, the network structure would become trivial compared with a null model. Such imperfectness in the adaptive behaviour of species provides an explanation for the empirical networks being slightly less nested or modular than the simulated ones, as seen in Fig. 2a of the main text. Yet, by such comparison, we can also conclude that the suboptimality in structure is marginal for most real networks cited in this study.

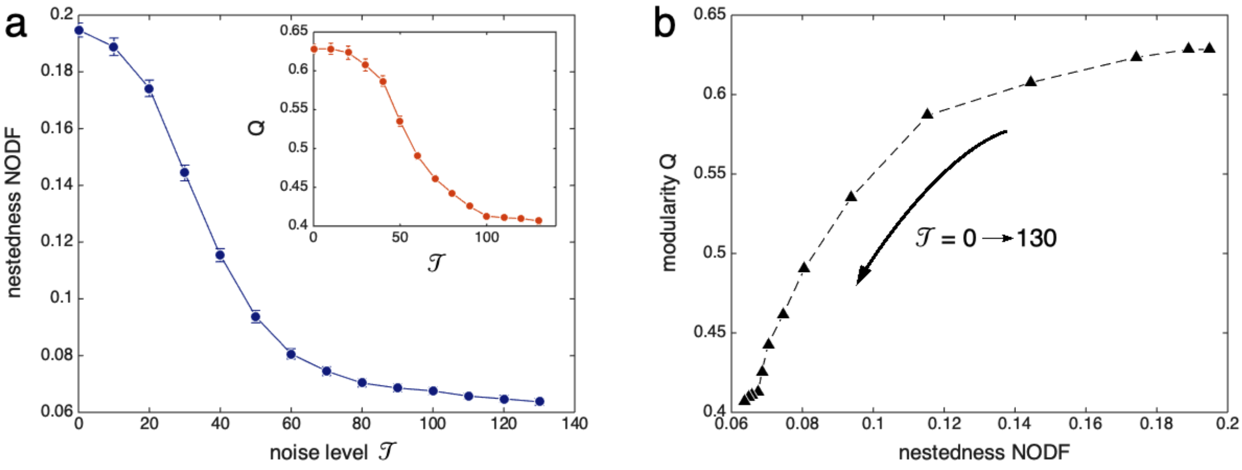

Supplementary Figure 27. Suboptimal structure under noise effect. Both nestedness NODF and modularity Q decrease with virtual temperature $\mathcal{T}$, as shown separately in **a** and in the dyadic $(Q, NODF)$ plane in **b**. The rescaling coefficient k_T used here is 0.0001. Data in panel a are obtained from 50 simulation runs and presented as mean values ± SD.

SUPPLEMENTARY REFERENCES

[1] Hutchinson, G. E. Concluding remarks. *Cold Spring Harbor Symp. Quant. Biol.* **22**, 415–427 (1957).

[2] MacArthur, R. & Levin, R. The limiting similarity, convergence, and divergence of coexisting species. *Am. Nat.* **101**, 377–385 (1967).

[3] Zhang, F., Hui, C. & Terblanche, J. S. An interaction switch predicts the nested architecture of mutualistic networks. *Ecol. Lett.* **14**, 797–803 (2011).

[4] Suweis, S., Simini, F., Banavar, J. R. & Maritan, A. Emergence of structural and dynamical properties of ecological mutualistic networks. *Nature* **500**, 449–452 (2013).

[5] Bastolla, U. & et al. The architecture of mutualistic networks minimizes competition and increases biodiversity. *Nature* **458**, 1018–1020 (2009).

[6] Wright, D. H. A simple, stable model of mutualism incorporating handling time. *Am. Nat.* **134**, 664–667 (1989).

[7] Bascompte, J., Jordano, P., Melian, C. J. & Olesen, J. M. The nested assembly of plant–animal mutualistic networks. *Proc. Natl. Acad. Sci. USA* **100**, 9383–9387 (2003).

[8] Scheffer, M. & van Nes, E. Self-organized similarity, the evolutionary emergence of groups of similar species. *Proc. Natl. Acad. Sci. USA* **103**, 6230–6235 (2006).

[9] Almeida-Neto, M., Guimarães, P., Guimarães, P. R., Loyola, R. D. & Ulrich, W. A consistent metric for nestedness analysis in ecological systems: reconciling concept and measurement. *Oikos* **117**, 1227–1239 (2008).

[10] Newman, M. E. J. Modularity and community structure in networks. *Proc. Natl. Acad. Sci. USA* **103**, 8577–8582 (2006).

[11] Flores, C. O., Poisot, T., Valverde, S. & Weitz, J. S. BiMat: a MATLAB package to facilitate the analysis of bipartite networks. *Methods in Ecology and Evolution* **7**, 127–132 (2016).

[12] Flores, C. O., Poisot, T., Valverde, S. & Weitz, J. BiMat. *http://bimat.github.io* .

[13] Ortega, R., Fortuna, M. A. & Bascompte, J. The Web of Life Dataset. *http://www.web-of-life.es* .

[14] Mucha, P. J., Richardson, T., Macon, K., Porter, M. A. & Onnela, J. Community structure in time-dependent, multiscale, and multiplex networks. *Science* **328**, 876–878 (2010).

[15] Bassett, D. S. *et al.* Robust detection of dynamic community structure in networks. *Chaos* **23**, 013142 (2013).

[16] Palla, G., Barabàsi, A. L. & Vicsek, T. Quantifying social group evolution. *Nature* **446**, 664–667 (2007).

[17] Saavedra, S., Reed-Tsochas, F. & Uzzi, B. A simple model of bipartite cooperation for ecological and organizational networks. *Nature* **457**, 463–466 (2009).

[18] Santamaría, L. & Rodríguez-Gironés, A. Linkage rules for plant-pollinator networks: Trait complementarity or exploitation barriers? *PLoS Biol.* **5**, 354–362 (2007).

[19] Guimaraẽs, P. R. & et al. Interaction intimacy affects structure and coevolutionary dynamics in mutualistic networks. *Curr. Biol.* **17**, 1797–1803 (2007).

[20] Fontaine, C. Abundant equals nested. *Nature* **500**, 411–412 (2013).

[21] Taper, M. L. & Case, T. J. Models of character displacement and the theoretical robustness of taxon cycle. *Evolution* **46**, 317–333 (1992).

[22] Gillespie, D. T. Stochastic simulation of chemical kinetics. *Annu. Rev. Phys. Chem.* **58**, 35–55 (2007).

[23] Newman, M. Clustering and preferential attachment in growing networks. *Physical Review E* **64**, 025102 (2001).

[24] Metz, J. A. J., Geritz, S. A. H., Meszéna, G., *et al.*, “Adaptive dynamics: A geometrical study of the consequences of nearly faithful reproduction,” in *Stochastic and Spatial Structures of Dynamical Systems*, van Strien, S. J. and Verduyn Lunel, S. M., Eds., 183–231, Elsevier Science, North-Holland (1996).

[25] Geritz, S. A. H., Kisdi, E., Meszéna, G. & Metz, J. A. J. Evolutionarily singular strategies and the adaptive growth and branching of the evolutionary tree. *Evolutionary Ecology* **12**, 35–57 (1998).

[26] Dieckmann, U. & Doebeli, M. On the origin of species by sympatric speciation. *Nature* **400**, 354–357 (1999).

[27] Loeuille, N. & Loreau, M. Evolutionary emergence of size-structured food webs. *Proc. Natl. Acad. Sci. USA* **101**, 5761–5766 (2005).

[28] Metropolis, N., Rosenbluth, A. W., Rosenbluth, M. N., Teller, A. H. & Teller, E. Equation of state calculations by fast computing machines. *Journal of Chemical Physics* **21**, 1087 (1953).

[29] Huang, K. *Statistical Mechanics* (John Wiley and Sons, 1987).
